# Pathology

**Published:** 1825-01-01

**Authors:** 


### PATHOLOGY.

Actio læsa suum designat singula morbum;
Preterea morbum partis mutatio signat.

1. *Melanosis.** In October 1822, our authors had an opportunity of examining a fine specimen of this very curious disease in the horse. The animal was emaciated, and seemed to have been long under the influence of the disorder. During life they saw a number of tumours, hard but moveable, under the root of the tail and in the neighbourhood of the rectum. Under the delicate mucous membrane of the verge of the anus, they were seen of a deep black colour. On the left buttock an enormous mass of the same was felt immediately under the skin, but connected with deeper parts. In the centre of this mass was an ulcerated opening, from which a fetid pus issued, mixed with small

* Messrs. Cullen and Carswell. Ed. Transactions, vol. i, p. 264.

pieces of the melanosis. On dissection, great masses of this substance were found in other places besides the cellular texture.

"There were many seated at the root of the neck, on the right side, corresponding to the subclavian region in man, partly in the cellular substance lying between the muscles, and partly in the very substance of these latter. There were a few near the base of the scapula, under the panniculus carnosus. Some were found in the groin, on the same side, in the seat of the superficial absorbent glands. We noticed one large mass, of the shape and size of an orange, between the six and seventh ribs of the right side, which had originated in the substance of the intercostal muscles, and had increased inwardly, so as to raise the pleura from the subjacent bone and muscular strata. The peritonæum had melanose matter lying upon it in streaks at that part where, leaving the bladder and rectum, it gives a covering to the last lumbar vertebra. The pleura in several places was similarly affected, and chiefly where it forms the mediastinum, and gives a covering to the pericardium. From near the termination of the aorta, down the lumbar region of the left side, into the pelvis by the side of the rectum, and out of it by the exterior aperture, there extended an immense chain, beginning above by one or two small insulated tumours, situated on the aorta, gradually increasing in bulk, till, at last, the black matter formed near the anus, masses as large as a child's head. From the anal region, the disease appeared to have spread into the muscles of the buttock. Such were the situations in which this curious disease was found, the substance of all the viscera being quite healthy." 266.

After a careful examination, our authors became satisfied that the large masses of melanosis were a congeries of smaller ones of different sizes. Each was surrounded by a cyst of condensed cellular membrane. These cysts did not communicate, but merely adhered together, thus forming large masses. Some of the individual cysts were no larger than peas, others as large as an apple, and of all intermediate magnitudes.—The investing cysts of the melanosis, situated in other structures than the cellular membrane, as in the muscular structure, were remarkably thin, so as, in some places, to be hardly detected. The tumours when cut into, were all of a deep black colour, like the pigmentum nigrum, or China ink. Most of them were as hard as cheese, while others were much softer. The vessels in their vicinity were not enlarged. Nerves were seen, sometimes running over the surface, sometimes traversing the very substance of the black masses, but without any cognizable change in the nerves themselves. The surrounding muscular texture was unchanged, and seemed merely separated in its fibres for the lodgement of the melanose matter.

"On the pleura and peritonæum, the disposition of the melanosis was somewhat different. It was effused on the surface of these membranes, not in round masses, but in streaks and patches. There seemed to have taken place on their surface a formation of cellular tissue, into which the black matter appeared to have been poured out, for it could be scraped off, and the serous coat was still left smooth and entire. The matter, too, was much softer, so as to be semifluid: the membranes upon which it was formed bore no traces of vascularity." 268.

This case they have thought proper to lay before the Society, because they think it well calculated to convey a general idea of Melanosis, even in the human subject. The striking appearances, as above recorded, are often met with in horses, constituting a peculiar disease to which the French Veterinary Surgeons have given the name of charbon, or maladie charbonneuse.

In the human subject, we occasionally find the disease equally well marked in its characters, and equally independent of other alterations of structure. Breschet has described cases of this kind: but the best specimen of melanosis, in the opinion of our authors, occurred lately in the clinical practice of Dr. Home, of which they are permitted to give an account.

*Case.* John Houston, aged 51, a shoemaker, complained on the 18th of March, of pretty severe symptoms of pleurisy, for which he was bled, blistered, and purged, with considerable relief. Up to the 23d, the symptoms diminished daily, the cough continuing, which, however, also gave way to purgatives, and pectoral medicines. On the 28th, an hæmorrhoidal affection supervened, and yielded to leeches. On the 1st April, he complained only of debility, for which a generous diet was ordered; but he died that night.

Here was a man submitted to the daily inspection of a discerning and experienced physician, as well as to the eyes of several intelligent pupils, without exhibiting "*any peculiar symptom that could have led to the suspicion of a deep-seated internal disorganization.*"

"On examination of the body thirty hours after death, the first incision exposed a number of black globular bodies, lying in the cellular tissue between the pectoral muscle, and third and fourth ribs of the left side, about the size of peas, and some of them adhering to the periosteum. The substance of the rib itself was completely black, as was also the sternal third of the clavicle of the same side; but these bones were in no other way altered from their natural state. Upon opening the chest, the pleura was found studded with similar tumours, here and there insulated, but in general aggregated, so as to resemble clusters of purple grapes. The last mentioned phenomenon was particularly remarkable on the right lung, along the bodies of the vertebræ on both sides, and upon the upper surface of the diaphragm on the left. Many of the tumours were hardly raised above the pleura, but others had long slender necks, so that they were like polypi. In colour most of them were jet black; others of a deep purple, or even reddish hue, while a third sort seemed to contain portions of a peculiar white colour, blended with black. *The lungs were extensively beset with these tumours; and several of an exceedingly small size were detected under the mucous membrane of the bronchiæ.* The pericardium, and the very substance of the heart were studded with melanose bodies. *As the cause of the symptoms under which the patient laboured during life, upwards of three pounds of fluid*

*were found in the cavities of the pleura*, and there was a thin pellicle of albuminous matter covering the surface of the lungs. In the abdomen, the liver, spleen, kidneys, omentum, and peritoneum in different places, were affected in the same manner; but the tumours in the liver contained a considerable portion of white cerebriform matter. To shorten the description, perhaps already too long, we may state in general, that similar tumours were found connected with the internal table of the skull, where they had formed for themselves little excavations; in the subcutaneous cellular substance of the thorax and abdomen, so as to be discernible through the integuments; and, lastly, among the fibres of the intercostal muscles. Doubtless, had the examination been pushed further, we might have found the disease in other organs, but from peculiar circumstances, it was impossible for us to render the dissection complete." 274.

When the reader peruses this dissection, and compares it with the passage just preceding, where our authors aver, that there were no symptoms to indicate hydrothorax and extensive disorganization of the lungs, he will be a little surprised. The stethoscope or percussion would have detected these lesions with the greatest ease, and therefore it is manifest that neither of these diagnostic measures were tried—a most culpable piece of neglect, which we have no reluctance in holding up to general reprobation. The physician who is placed in a public situation, as in an Hospital or Infirmary, betrays his trust and neglects his duty, when he obstinately refuses to put in action the improvements which take place from time to time in the science he professes. We hope these remarks will have a proper effect on those whom they concern.

The tumours found in the different parts of the body possessed certain general features. They consisted of a cyst separated by a loose cellular tissue from the surrounding textures, and containing the peculiar black matter of the disease in question, varying much in the degree of consistence, from quite solid to nearly fluid. In the liver only there was a remarkable variety noticed. The tumours here were as large as chesnuts, some of them white, consisting of cerebriform matter—others pure melanose—and others still containing a mixture of both.

Another excellent specimen of melanosis presented itself in the practice of professor Alison. The patient was a female, aged 42 years, admitted into the Royal Infirmary on the 3d of June, complaining of severe pains shooting down from the loins to the inferior extremities, and round the abdomen. She had similar pains in the right shoulder and arms, increased at night, and by motion. She had become weak and emaciated since her complaints began, and was liable to shivering, followed by flushing and perspiration. The abdomen was swelled, but did not fluctuate on percussion, the distention varying in degree at different times of the day. She had thirst, with scanty, high-coloured urine, and a hard moveable tumour could be felt in the iliac and hypogastric regions. She was also liable to paroxysms of dyspnœa during the night. She stated her complaint to be of five or six weeks standing, and to have commenced after exposure to cold, with pains in the joints.

*Treatment.* Leeches, fomentations, anodynes and diaphoretics.—Tympanites occurred on the 7th June, for which calomel and opium were employed, and continued till the 9th, when soreness of the mouth was evident. The pains of back and lower extremities increased, with distention of the abdomen, all aggravated by globus hystericus. On the 21st, several small painful tumours were observed on the integuments of the abdomen. Examined by a skilful accoucheur the internal tumour was pronounced to be unconnected with the uterus. On the 1st July, great debility, with hectic fever, and tendency to sloughing of the sacrum. On the evening of the 7th, after vomiting a dark-coloured matter, she soon died.

*Dissection.* There were several dark-coloured tumours dispersed over the body, especially in the mammæ. They were encysted in the cellular substance, and contained melanose matter of soft and pulpy consistence. Within the abdomen most of the cellular and adipose textures had disappeared. The peritoneum lining the parietes was of a blackish colour, and the black matter was irregularly deposited in striæ and spots upon the inner side of the membrane, which had lost much of its transparency. The omentum presented a similar appearance. Between the folds of the mesentery, and beneath the serous membrane of the intestines there were numerous black spots and small tumours. The external surface of the ovaria had a dark, shining, lobulated appearance, with black matter irregularly deposited in spots, giving a mottled appearance to the whole. When cut into, their substance was uniformly black. This melanose matter was found in various other portions of cellular texture throughout the thorax and head. Even the bones of the sternum and some parts of the scull were black. The kidneys, liver, spleen, and mucous membranes were free from the disease.

Our authors here make several observations on the distinction between the disease under consideration and cancer and fungus hæmatodes. We think it is abundantly evident that there is a striking difference between melanosis and these last-mentioned diseases. Scirrhus, for instance, chiefly attacks glandular structures that have been previously in a state of inflammation—while hæmato-fungoid tumour attacks every kind of texture. They both begin in a definite point—gradually extend their ravages in every direction—convert all the proximate tissues, by a kind of assimilative process, into their own kind of matter—and finally prove fatal, by sloughing, hæmorrhage, or irritative fever. In their course, they are generally attended with lancinating pains, which is seldom the case in melanosis. The matter of the latter was constantly found in cysts, which our authors consider as one of its distinctive characters.—Neither in melanosis had the neighbouring textures of the body undergone any alteration in consequence of the ravages of the disease. The bones were coloured, but not diseased. The parenchymatous viscera were not altered in texture. The substance adjacent to the cyst was as healthy as the rest of the organ. There were no traces of vascularity. These and other considerations induce our authors to believe that melanosis, in its simple form, is of a nature quite distinct from cancer, both in its seat, symptoms, progress, and anatomical characters. With this negative kind of knowledge we must be satisfied, at present, till the disease shall have excited more generally, and for a longer time, the attention of the medical world.

---

2. *Large Biliary Calculus.** Dr. Craigie has recently narrated the case of an old lady, who voided a large biliary calculus, preceded by symptoms of ileus, and has come to such extraordinary, and, as we think, such erroneous pathological conclusions, that we shall give the particulars of the case, and our reasons for differing, *toto cœlo*, from the respectable author.

On Sunday, 28th March, an elderly lady, after walking from Leith to Edinburgh, became sick,—vomited,—and felt considerable diffused pain in the belly. These symptoms got more severe on Monday (29th) and also on Tuesday. The bowels had not been opened since Saturday the 27th, although repeated purgatives had been administered and vomited up again. Blood was taken from the arm on the 30th, (Tuesday), and on Wednesday, 31st, Dr. Craigie first saw the patient. She had now frequent painful retchings and occasional hiccup—" vomiting at intervals of a *yellow coloured fluid*, of the *bitter* taste of which she complained much." The tongue was dry and brown—there was much thirst—skin parched and imperspirable—face and extremities inclining to be cold—pulse 86 and small—whole surface of the belly extremely tender and painful, with some tension—complaining most of pain in the line of the transverse arch of the colon. Dr. C. directed 15 or 20 ounces of blood to be taken, a purgative injection to be thrown up, and the patient to be put into a warm bath. A purgative and anodyne pill was also administered by the mouth. In the evening she was again bled, and 40 drops of laudanum ordered on removal from the bath. *Thursday, April 1st.* Had a few hours sleep and ease in the night; but at six in the morning the pain returned wiih great violence, " the sickness and vomiting of *bilious matter* almost incessant." No passage through the bowels. Eighteen leeches to the abdomen—turpentine and assafœtida injection—fomentations. The injection returned without a particle of feculent matter. An opiate at bed-time. *Friday, 2d April.* Abdominal pain is not relieved—vomiting recurs at intervals—bilious matter ejected is darker, and " seems accompanied by minute portions of fæcal matter"—bowels obstinately bound. A tobacco enema exhibited, and remained in the bowels from 12 till four o'clock. A senna enema was then thrown up and brought off some of the turpentine injection. Another tobacco glyster was thrown up, and Dr. Craigie left the patient in such a wretched state that he expected to hear of her death every moment. *Saturday, 3d April.* (stated by Dr. C. to be the 11th) there was a sensible improvement. The

---

* Dr. David Craigie. Ed. Journal, October, 1824.

vomiting had not returned since the use of the bath the night before—she had slept a good deal. The tobacco injection was repeated, as the bowels had not now been opened for a week. *Sunday, 4th April.* Still easier—very slight traces of feculence in one of the injections. An injection of tobacco being this day thrown up, the patient soon began to experience a sensation of intestinal motion, and desire for the bed-pan—on which "she voided, with a feeling of acute pain, some hardened excrement—uttered a loud shriek, and instantly fell from the nurse's arms, without giving the slightest sign of consciousness." On recovering from this, the motion was examined, and a globular biliary concretion was found, weighing 160 grains, and being *one inch and two lines* in diameter. It is hardly necessary to say that the woman passed considerable quantities of fæces after the expulsion of the gall-stone, and soon recovered.

Now what does our author conclude from this case?—He concludes that this gall-stone passed through the cystic duct and ductus communis during this week of illness, and produced all the symptoms of ileus, there being no obstruction in the intestinal canal all the time! Be it remembered that this patient presented not the slightest trace of jaundice while the duct was plugged up with this concretion—nor did she fail to vomit up bilious matters almost all the time of her illness. This, Dr. Craigie thinks, is rather wonderful—and truly so it would be, if the concretion had been passing along the ducts. Dr C. thinks that, from the moment it got into the intestinal canal there could be no obstruction to its discharge from that tube. But we beg to inform him that, to our knowledge, a physician in this metropolis lost a patient within these few years, in consequence of a biliary concretion, not larger than a pigeon's egg, becoming jammed in the ileum and causing complete ileus, terminating in death. Is it not evident to every reflecting mind that this immense concretion passed through an ulcerated passage from the gall-bladder into the duodenum or other small intestine, and then produced vomiting, constipation, and all the other symptoms till finally expelled from the rectum? How else should the bowels be obstinately confined all the time—how could she continue to vomit up bile every day, if the ducts had been plugged up—how could she have been free from jaundice? Among the cases quoted by our author, the one published by Mr. Brayne in the 12th volume of the Medico Chirurgical Transactions (and in our Journal for March, 1824, Number 16) might have opened his eyes—for it was precisely in point. A woman, after much suffering, passed a large biliary concretion *per anum*, and recovered; but dying afterwards of another disease, she was examined, and an adhesion was found between the gall-bladder and duodenum the breadth of a shilling, with an aperture in its centre. What can be more clear than that it was by such a passage, and not by the biliary ducts, that the concretion made its way in Dr. Craigie's case. He saved his patient's life, in all probability, by the tobacco enemata, by which the irritated and spasmed intestines were relaxed, so as to permit the concretion to pass along. We think, on mature reflexion, Dr. Craigie will admit that his pathological conclusions have been too hastily drawn—and that he has viewed the case in a wrong light. At all events, we appeal to the public as the ultimate judge.

---

3. *Occult Disease of the Chest.** There is no other organ in the body, we think, which will appear to carry on its function so regularly as the lungs, when in a state of extensive structural disease. It would seem almost that a man may breathe tolerably well, under ordinary circumstances, with a few cubic inches of sound lung.

Dr. Carter admitted a man, 50 years of age, into the Kent Hospital, labouring under excessive debility, with a dry furred tongue, frequent feeble pulse, tremor, and complaining of great pain in the right thigh and knee, which were swelled. He made no complaint of any thoracic affection till the day of his death, about a week afterwards; nor did there appear any symptom of disease in that quarter. On dissection, the pleura pulmonalis and costalis were found adherent on both sides. The right lung was diseased throughout, being studded with tubercles—some suppurated, some crude. There was also calcareous matter in cysts. "There was universal inflammation of the lungs and of the pleura lining the ribs on the right side." There was also caries of the bodies of two of the dorsal vertebræ. The cavity of the chest contained a considerable quantity of fluid. The abdominal viscera were sound.

---

4. *Inflammation of Veins.*† On the evening of 19th of March, 1823, Dr. Duncan was summoned to Mr. A. a medical gentleman, 88 years of age, whom he found in bed, with his left hand swollen, red, and painful, as if affected with *erysipelas phlegmonodes.* There was a sore on the knuckle of the fore-finger, resembling the remains of a blister or small boil. There was also some swelling, though inconsiderable, of the wrist and fore-arm. His countenance appeared livid, his respiration was frequent and interrupted, pulse 120 and full, tongue furred, thirsty. He could give no information respecting the sore on his knuckle. He appeared distressed and agitated, but not alarmed. Dr. D. was aware that the patient had recently been much harassed; and he ascribed great part of his present illness to moral causes. Dr. D. afterwards learnt that Mr. A. had dined out on the preceding Saturday, in good health, but on Sunday evening complained of being cold. On Monday his landlady observed a boil on his knuckle, which he opened with a lancet that had been employed a few weeks previously in opening a carbuncle. During the winter he had studied hard, sitting up late and rising early. Poultices, and an aperient. 20th. Apparently much better—pulse down to 80, and without febrile irritation—affection of the hand subsi-

---

* Dr. Carter. Med. Repos. October, 1824.

† Dr. Duncan, Jun. Med.-Chir. Trans. of Edinburgh, vol. i. p. 439, et seq.

ding. In the evening, however, there was a great exacerbation—pulse 120, not full—swelling extending slowly up the arm, though without pain. 21st. No remission, as expected, this morning. Had passed a restless night, and taken some laudanum. The swelling was still making progress up the arm, but unattended with redness or pain.—*Evening.* Dr. D. received a hurried message. The patient had sent for Dr. Kenney and Dr. R. Hamilton, the latter to bleed him, as he had some hæmoptysis during the day. He was now looking extremely ill—the sore appeared irritated—the hand and fore-arm much swelled, and one or two red streaks running up from the elbow to the shoulder, in the course of the cephalic vein. His respiration was now again much affected, but he denied having any pain in the chest. He had frequent cough, but little expectoration, the sputum being tinged with blood. Pulse 110, not strong—thirst—foul tongue. Sixteen or eighteen ounces of blood were taken from the arm, and proved buffy, and cupped. The pulse fell to 96, but no other salutary change was perceptible. He took some light supper with a relish. In the night Dr. H. was again sent for, on account of wandering pains in the stomach, from flatulence. 22d. All the symptoms aggravated—pulse 120—swelling extending up the arm—no swelling of the axillary glands. Twelve leeches to the arm and then an enema thrown up. *Evening.* Decidedly worse—pulse 120—subsultus tendinum—transient delirium—made no complaint of pain—expressed no apprehension of the result. A full dose of laudanum. The early part of the night was restless and agitated. At two in the morning of the 23d. a heavy wandering, and a sinking state took place. At four in the morning Dr. H. found him apparently dying, the blood still oozing from the leech-bites—pulse 140, and very feeble. Heat, stimulants, &c. were tried in vain. The breathing became sonorous and oppressed, and death soon closed the scene.

*Dissection.* The skin and muscles of the chest were healthy—general adhesion of the lungs to the costal pleura, pericardium, and diaphragm, by recent exudations of coagulated lymph. There were several spots of condensed livid lung, one of them, larger than the rest, seemed as if affected by gangrene, but had no bad smell. In the left side of the chest there were five or six ounces of brown-coloured fluid. The lungs did not collapse when freed from their adhesions, but remained full and tumid. Yet the lungs were crepitous, though considerably gorged with serum, especially the right. Several tubercles were found, and some calculous depositions. The other viscera sound.

"Many livid spots were observed externally on the left arm; and on the back of the hand of the same side, between the heads of the metacarpal bones of the first and second fingers there was an ulcer, around which there was an evident swelling of the cellular substance, extending more or less up the whole arm. On making a long incision from this ulcer to the top of the shoulder, a small abscess seemed to be laid open at the bend of the arm, but it proved to be the cephalic vein which had been divided, and was full of purulent matter. The vein was not traced with more care, which, from its increased size and solidity, was easily done, and we found that the veins coming from the back of the fore-finger, middle and ring fingers, were all diseased; but that from the little finger was healthy. The morbid state of the vein extended upwards along the whole course of the cephalic to its termination in the left subclavian, which remained perfectly healthy. The disease of the vein consisted in external redness, arising from the increased size of the *vasa vasorum;* thickening of all its coats, so that it remained like an artery, round without collapsing; increased size, especially in the fore-arm; its containing no blood in any part of its course, and being generally filled with purulent matter, except in a few places, where it seemed empty; and in the inner coat being every where red and thickened. On the back of the hand, it appeared as if matter had been formed in the cellular texture itself; but upon more minute examination, it was quite evident that it existed solely within the veins. Throughout, the cellular and muscular texture of the arm were almost healthy and natural, even in the immediate vicinity of the diseased parts of the vein; but towards the end of the dissection, a considerable quantity of serous fluid was observed to have escaped from the divided tissues." 448.

We think Dr. Duncan is entitled to consider the above as a case of "death solely from inflammation of the vein," although there were peculiarities in the case which are not to be entirely overlooked. There was evidently considerable disorder of the lungs, both recent and of long standing—and it is improbable that the patient could have survived many years, from the progress of the tubercles. Whether this condition of the respiratory organ had any effect in producing or accelerating the fatal event, it is difficult to say. Dr. Duncan was struck, even at his first visit, with the livor of the patient's countenance, and the disordered state of his breathing. Still the immediate cause of death cannot be fairly attributed to the pulmonic affection. On the other hand, it is probable, as Dr. Duncan observes, that if the recent pulmonic affection existed previously to the fever produced by the venous inflammation, it would be aggravated, as all local complaints are, when a general excitement of the system supervenes. As to the sudden termination of the disease, experience has shewn but too certainly, that inflammation of a vein, when considerable, often destroys life in eight or ten days. The nature of the local affection was evidently different from erysipelas of the cellular texture. There was an absence of pain on pressure, except along the course of the vein—there was no great inability of moving the limb—and, lastly, the local symptoms were comparatively slight. All of these, our author thinks, tend to distinguish the disease from erysipelas. Still there was increased action in the cellular tissue of the whole limb, with a corresponding effusion, not of pus or coagulable lymph, but merely serum. The consequent fulness and extension of the limb assisted in preventing the medical attendants detecting the real nature of the disease. During the life of the patient, and indeed previous to incision, there was little appearance, Dr. D. observes, of the vein being the chief seat of the disease, if we except the red streaks running up the arm, and the tenderness over the immediate track of the vessel. There was no longitudinal ridge or cord-like substance distinguishable.

In respect to the actual exciting cause of the venous inflammation, there can be little doubt that it was connected with the phlegmon on the knuckle—which phlegmon was, in all probability, produced by some virus on the lancet, by which it was opened. Dr. Duncan thinks, however, that this effect would not have been produced under common circumstances. He considers it probable that, in opening the boil, the gentleman had unfortunately penetrated a vein situated beneath it:—"and perhaps it is as probable that he had inoculated himself with matter from his own body (from the boil) as that the lancet, which he had wiped apparently clean, should have remained so long contaminated."

---

5. *Dissection of Lord Byron.* A foreign Journal (*Gazette de Santé*, 25 August, 1824) gives the following minutes of the *post mortem* examination of our illustrious countryman and poet—Lord Byron. From this dissection, it is manifest that his Lordship lost his life, in all human probability, by his inveterate prejudice against blood-letting.

1*mo.* The bones of the cranium were extremely hard, and without any trace of suture or diploe, resembling, in that respect, the skull of an octogenarian.

2*ndo.* The dura mater was so firmly attached to the internal surface of the cranium as to be separated by great force only. The vessels of this tissue were much injected. It was adherent, in several places, to the pia mater.

3*tio.* The vessels of the medulla oblongata were finely injected with blood, and the part itself red and swelled. There were about two ounces of sero-sanguineous fluid in the different ventricles.

4*to.* The medullary substance of the brain greatly exceeded its usual relative proportion to the cortical, and was of very firm consistence. *The cerebrum and cerebellum weighed six medicinal pounds!*

5*to.* The lungs were sound, but of a gigantic size—"d'une grandeur presque gigantique."

6*to.* About an ounce of serum in the cavity of the pericardium. The heart was larger than ordinary, and its muscular substance relaxed.

7*mo.* The liver was smaller than usual, as were also the biliary ducts and gall-bladder.

8*vo.* The kidneys were large and sound.

From this report the editor of the Journal abovementioned concludes, that "a small bleeding at the beginning, or a very copious one at the height of the disease, would have saved his Lordship's life—but he obstinately refused to consent to the operation." This may be true; but we do not clearly see the foundation of the editor's next conclusion, namely, that Lord Byron could only have lived a few years more at any rate—"à cause de son excessive susceptibilité qui le rendait lade aisément, soit à cause de la violence de ses passions, de son travail excessif, soit à cause de sa negligence à employer des remèdes contre son extrême constipation." But M. Miguel ought to know that the passions (excepting *avarice*, to which his Lordship was not prone) subside with advancing age—that the labours of the intellect slacken with the passions—and that we very frequently see more attention paid to *health* and the preservation of *life*, as the one becomes enfeebled and the other draws towards a conclusion. We can perceive nothing therefore in Lord Byron's physical organization, to preclude the prospect of long life, in the absence of those accidents to which the best constitutions are liable.

---

6. *Memoir on the Inflammation of Nerves.* By L. Martinet, M.D. [Revue Medicale, June 1824.]

Considerable diversity of opinion obtains among pathologists and practitioners respecting the nature of neuralgic affections—some attributing the pain to an inflammation, more or less acute, of the neurilema of the nerve, while others consider it as a morbid state of the sensibility of the nerve, and independent of any inflammatory condition. Hitherto facts have been wanting in number to clear up this point. M. Martinet has directed much of his attention to this subject for ten years past, and the result is, that in comparatively few instances, is there actual inflammation or other organic change in painful affections of these parts. The neuralgiæ, on the other hand, depending on pathologic modifications of the sensibility in the nervous pulp, are very common. It is to the former or more rare class that the present paper is dedicated—namely, to diseases of the nerves depending on changes in structure, as tumours, erosions, inflammations, &c.—but particularly to inflammation, as the title of the paper expresses.

The seat of this neuro-phlegmasia our author has always found to be in the neurilema—nor does he know of any well authenticated case of inflammation in the nervous pulp itself. The cases which he relates, will, he thinks, establish a distinction in symptoms between the pure neuralgiæ and the inflammation of nerves. To these cases we shall now proceed.

*Case* 1. This case is incomplete, but still it bears sufficiently on the point under investigation. M—— was seized in the spring with a pain in the left arm, following the track of the cubital nerve, but not at first preventing the motions of the member. In a few days, the pain became so violent that the patient could not obtain any rest by day or by night. The cubital nerve swelled to a considerable size—equal to the little finger, and felt like a tense cord along the arm. No ease could be procured from any application, except from the immersion of the arm in warm water. This state of suffering lasted four months, the cubital nerve continuing all that time in the same swelled, rigid, and painful condition.

The author reasonably infers, that the symptoms of inflammation in the nerve here, were nearly as convincing as if the arm had been dissected. The pain was *permanent* too, which is a distinction between the neuralgiæ and the *neuro-phlegmasiæ*, if we may be allowed the expression. In the latter there may be remissions, but no clear intermissions.

*Case* 2. A man, of good constitution, had undergone great military fatigue, and exposure to cold for six months. On the 4th August, 1812, he was seized with acute and lancinating pain at the bend of the right arm, and following the track of the median nerve. This pain ceased suddenly, and the following day was transferred apparently to the right knee, and along the internal saphena nerve. It continued a week in this part, and gradually disappeared. Three days afterwards it fixed itself in the *left* arm, on the inside, extending from the arm-pit to the little and ring fingers. The pain daily increased, and the fingers above-mentioned were occasionally convulsed. Ou the 20th August the patient entered the Hotel Dieu. The arm was now extremely tender to the touch, and the patient could hardly move it. The track of the pain was from the arm-pit along the inner side of the arm to the elbow, where it traversed round the internal condyle of the humerus, extending along the radius and terminating in the fingers aforesaid. The cubital nerve could be felt enlarged and tense, especially between the elbow and arm-pit. The member was a good deal wasted, though it preserved its sensibility. The little and ring fingers were now paralytic.

*Case* 3. Dubuisson, 49 years of age, subject to piles from the age of 31, enjoying habitual good health, became affected, four months before he entered the Hotel Dieu, with pain exactly similar, according to his own account, to the case last detailed. He attributed his complaint to a suppression of the hæmorrhoidal discharge, and exposure to damp and cold. The hæmorrhoids had been suddenly suppressed on the 10th June, when an acute pain took place from the shoulder to the fingers' ends of the right arm. This continued till the 17th, when he entered the hospital. The pain was now excessive, attended with a sense of formication, and an exacerbation every night. The radial nerve was felt tense and somewhat enlarged. When pressed, the increase of pain was felt along the whole course of the nerve. He could not move the arm. No redness or swelling of the integuments. The pulse was full and quick, the skin hot. A dozen of leeches along the nerve, followed by a poultice with laudanum. Next day, pressure on the nerve produced no increase of pain. The same treatment. The pain gradually decreased, and the patient was well at the end of a fortnight from this time. The symptoms and the result prove, our author thinks, that the nerve was inflamed, and that the case was not simply Neuralgia.

*Case* 4. A woman, 43 years of age, having always enjoyed good health, although the menstrual discharge had ceased at 22, began to feel pain along the left arm, in March 1810. This pain gradually increased, and became violent—extending from the shoulder to the fingers. A perpetual blister gave some relief, while kept open. But being healed, the pain along the inside of the arm became excessive, attended with numbness, sense of formication in the hand, and great increase of pain on pressure. In eight days after this, she became affected with pain in the other arm, and in the same direction. These pains continued, with more or less intensity, till the November following, notwithstanding various means were put in force to subdue them. She was ultimately worn out, and died on the 10th of the above-mentioned month, having been for some time paralytic of the upper extremities.

*Dissection.* The spinal arachnoid was red throughout a great portion of its extent, but the spinal marrow itself was sound. The median nerve of the left arm, for two inches after its separation from the plexus, was of a deep red colour, internally as well as externally. The anterior branch of the seventh cervical, which goes to the formation of the median nerve, was also red, but not so much so as the median itself. The other nerves of the arm were in their natural state. The brachial plexus on the right side presented nothing particular.

Case 5. A refractory conscript, of athletic constitution, after being chased closely through the woods, was at length seized by some Gens-d'armes, covered with perspiration, and in a state of delirious excitement. On the third day, this young man could not stand on his feet, nor could he stretch out his lower extremities. Excruciating pains were felt along the posterior part of the thighs, in the line of the sciatic nerves. The least attempt at motion aggravated these pains, and caused the patient to cry out. Inflammation of the lungs having supervened, this unfortunate youth died on the eighth day after the pains had commenced in the thigh.

*Dissection.* Passing over the state of the lungs, the sciatic nerves on both sides were enlarged to the size of the fore-finger, being also hard and resisting. Each filament of these nerves was distinctly visible, and separated from each other by a sero-sanguineous infiltration into the cellular tissue that unites them. They were penetrated by a multitude of small vessels which gave the red appearance to the nerves. There can be no doubt here of the inflammatory condition of the sciatic nerves.

Case 6. A man of the name of Faucha, 47 years of age, entered the Hotel Dieu, affected with congestions of blood about the head, for which he was bled, and the symptoms were relieved. Three weeks afterwards he complained of pain along the posterior part of the thigh, with which he had been afflicted at times before. This pain became excruciating. He attributed it, on this occasion, to cold he had caught the evening before. A blister gave no relief. The pain prevented, almost entirely, any motion of the limb, and was greatly exasperated by the least pressure made in the course of the sciatic nerve. The pain was constant, and of nearly the same degree of intensity. Fever supervened, and became every day increased. On the sixth day from the commencement of the sciatica this man fell into a profound coma, and died three days afterwards.

On dissection, much blood was found extravasated into the lateral ventricles and the septum lucidum torn. The sciatic nerve, which was the seat of the pain, was somewhat larger than the other, and of a red colour from the hip to the ham. At the superior part of the thigh the nerve was enlarged, for two inches, to a very considerable thickness, and in this part it was of a violet redness. Blood was infiltrated between its filaments, and could be squeezed out from thence. Cut across, the nerve was equally red in the anterior as in the exterior, and was much softer at this part than in the other portions.

*Case* 7. A man, 68 years of age, entered the Hotel Dieu, in the month of January 1817, afflicted with inflammatory affections of the abdomen and also of the head. On the 19th February, he was seized with sudden and violent pain in the ischiatic region, extending someway down to the thigh. Blisters and repeated applications of leeches afforded little relief. On the fifth day from the invasion of the sciatica, the patient died of the cerebral disease. On dissection, arachnitis was found to obtain, and some organic changes in the abdomen. The sciatic nerve was then examined. It was sound from its origin till it emerged from the pelvis. For three inches in extent it was found infiltrated with purulent matter, which penetrated between its minutest fibrils. The surrounding cellular tissue was also infiltrated with the same.

*Case* 8. A child, 12 years of age, who had long been affected with symptoms of pulmonary phthisis, complained of lancinating pains in the thigh, proceeding from the ham up to the great trochanter of the left side. The next day brought a great increase of the pain, and in ten or twelve days the child was unable to move the limb. Pressure on the course of the sciatic nerve occasioned a numbness and pain throughout the whole limb. No cause could be traced of this disease. Every evening there was an exacerbation of the sufferings, but no distinct intermission. Leeches were twice applied, and each time with relief. The patient remained somewhat stationary and rather better as to the pain for about two months. The pulmonary affection, however, made progress, and death took place from that cause. On dissection, the lungs were found tuberculated, and several of the tubercles were in a state of suppuration. The left sciatic nerve was not sensibly increased in volume, but at its upper part it was bathed, for a considerable space, with a sanious pus, and completely separated from the surrounding muscles in several points. Between its fibrils some sanious pus was also found. In this part the vessels of the nerve appeared injected. In its other portions it was of its natural white colour.

This case was communicated to Dr. Martinet, by Dr. A. Goupil, who has long been prosecuting the study of infantile diseases.

*Case* 9. A man, 44 years of age, had been affected for eight days with pain in the course of the right sciatic nerve, which had commenced soon after exposure to cold when in a state of perspiration. The pain had daily increased in intensity up to the time he entered the Hotel Dieu. When received, it was found that the pain was increased by cold and diminished by heat. The least motion exasperated the patient's sufferings. Pressure on the sciatic nerve caused a numbness in the whole limb, and excruciating pain in the point compressed. The temperature of the limb was increased to the feelings of another person, and appeared burning to the patient. There was some slight pyrexia, generally augmented in the evening. The warm baths only were employed. One day he exposed himself to a chill after coming out of the bath, was seized with peritonitis, and died twelve days after he came into the Hospital.

Dissection shewed the crural nerve increased to double the natural size for an inch and a half after its emergence from the pelvis. It was of a violet colour, and sprinkled here and there with spots of ecchymoses. The cellular tissue connecting the nervous filaments was injected and evidently inflamed. Above and below the point of lesion the nerve was in a natural state.

*Case* 10. A man, 56 years of age, had had a consecutive luxation of the head of the femur on the left side, and was in the Hotel Dieu for some months, when, on the 20th April, 1817, he was suddenly seized with such a violent pain in the thigh as to make him cry out. This pain was situated towards the ischiatic notch, and extended along the under side of the thigh and peroneal side of the leg to the malleolus externus. The slightest pressure on the course of the sciatic nerve, or the least motion of the limb caused considerable pain. Moxa was applied, but without relief, and various other measures were put in force with the same want of success. The pain continued unabated—fever became established, and the patient died on the 16th of May following. On dissection, the parts about the hip-joint were found inflamed, suppurated, and some of them quite disorganized. At its emergence from the sciatic notch the sciatic nerve appeared of a brown colour, for the space of two inches, both externally and internally. Its density, at this part, was considerably diminished. There was no change in any other part of the nerve.

*Reflections.* From the preceding cases it will have been observed, that, in every instance where dissection was performed, and the parts brought into view, redness, more or less, of the nervous tissues was found—evidently dependent on injection of the vessels of the neurilema or membrane investing the pulp of the nervous fibrils. Partial ecchymoses, or sero-sanguineous infiltrations were also generally present, and sometimes traces of suppuration. In most instances the nerves were augmented in volume—but rarely softened in structure. The symptoms accompanying these lesions of the nerves consisted in more or less complete diminution of their functions—in pain, either of a lancinating or benumbing kind—never suddenly intermittent, as in the neuralgiæ sine inflammatione—but generally continued, with occasional exacerbations and remissions. These symptoms are sufficiently characteristic of the neuro-phlogoses, which are much less frequently observed, however, than the mere neuralgiæ, or morbid alteration of nervous sensibility, without inflammation. The treatment of the former class must be clearly antiphlogistic.

---

7. *Perforations of the Alimentary Canal.** Many writers have noticed this pathological fact—and we all know the controversies that have been carried on respecting the cause of the phenomenon—some referring it to a living, some to a *post-mortem* origin. It is the object of Dr. Gairdner, however, to draw the attention of his brethren to some cases of infantile disease, remarkable on account of some very singular appearances on dissection—namely, perforations or erosions of the coats of the stomach—the former resembling those described by John Hunter, and attributed by him to the action of the gastric juice after death. We shall first give an abstract of the cases.

*Case* 1. Dr. G. was invited to the dissection of a child, on the 8th August, 1821. He learnt that it was 13 months old, and had been troubled with cough from the preceding January. About three weeks before death, the child became feverish, with a pulse at 160—respiration 70 in the minute, but without much heat of skin, which indeed was sometimes colder than natural. There was much expectoration with the cough, and alternate perspirations and diarrhœa. There was also occasional vomiting. Among the matters ejected by vomiting there was generally a quantity of purulent expectoration previously swallowed.—The child could not lie on its left side for three weeks—there was a swelling perceptible in the left hypochondrium. It was unable to suck—had an insatiable thirst—slept much, though easily disturbed—and had greenish evacuations from the bowels during the last three weeks of its life.

*Dissection.* Lungs much diseased, and full of tubercles on both sides, with abscesses on the left side. The contents of the stomach were extravasated into the abdominal cavity through a large opening in its left extremity near to its natural connexion with the spleen. The edges of the opening were ragged, soft, and extremely thin, with a slightly reddish tinge all around the perforation, forming, as it were, a narrow border, not a tenth of an inch in breadth. With this exception, there was no appearance, either in the stomach or neighbouring parts, indicative of vascular turgescence or inflammatory action. The opening was large enough to admit four fingers.

---

It is evident enough, as our author observes, that in this case, the pulmonary complaint was the primary disease, and this singular state of the stomach recently conjoined with it.

Of the next two cases (both in the same family, and both fatal) one was not examined after death, and therefore we are far from being so confident as Dr. Gairdner that the disease was perforation of the stomach. The child had been weaned nine days previously to its death. For seven days it had diarrhœa, and for the last three days, vomiting. "Coldness of the surface and extremities, a feeble, fluttering, irregular pulse, together with considerable emaciation and extreme weakness, preceded the fatal termination." However similar these symptoms may have been to those presented by children who died with perforation of the stomach, we certainly are not warranted, in the present state of our knowledge on the subject, to come to Dr. Gairdner's conclusion, without the demonstration of *post-mortem* inspection.

*Case* 3. A brother of the preceding, nursed by his mother, had enjoyed good health, and had every appearance of thriving till it was eight months old. At this time it was suddenly seized with a fit of violent crying—a thing very unusual with it. From that period the mother thought it looked less healthy, which was probably owing to a state of dentition. He was weaned at the end of eleven months, and on the day of weaning, was troubled with a trifling degree of diarrhœa. This went on, in a light manner, for a week, without any medical advice. On the evening of the 8th of June he had a powder, consisting of one grain of calomel and three of prepared carbonate of lime, which was repeated on the 9th, and operated with considerable power on the bowels. On the 10th, the bowel-complaint still continuing, he had a powder of carbonate of lime, rhubarb, and Dover's powder. 11th. Vomiting, and continuance of diarrhœa. A quarter of a grain of opium in a draught. 12th. Stomach still irritable—diarrhœa checked in the night, but returned in the morning. Emaciation and debility very evident. Pulse natural, as from the beginning—no heat of skin, or other febrile symptom. 14th. Languor and exhaustion to a great degree—ardent thirst—retchings—unrefreshing slumbers. 15th, aphthous specks on the tongue, but no ulcerations. The diarrhœa and emaciation progressively increased, and the child sunk on the 17th, or seventeen days from the first occurrence of vomiting. During the last two days of the infant's existence the stools consisted of mucus mixed with bile, and occasionally streaked with blood.

*Dissection.* "On opening the abdomen the viscera appeared at first sight to be all sound; but on slitting up that part of the omentum which joins the great curvature of the stomach to the transverse arch of the colon, the contents of the stomach were seen pouring out through several perforations in that viscus. These perforations were four in number; they were situated on the posterior surface of the stomach, not far from its splenic extremity; they were nearly of the same size, the largest scarcely big enough to admit the point of the fore-finger. They were separated from each other by portions of the substance of the stomach, not so broad as the apertures themselves, and these portions were in an exceedingly soft state, and very thin. More of the internal coats appeared to be destroyed than of the peritoneal. No vascular turgescence, no adhesions, nor other marks of inflammation were discoverable. The gall-bladder was full of bile. The intestines appeared thin and semi-transparent, partially distended with air, and strongly tinged, in one or two places, by the transudation of bile: they contained scarcely any solid or fluid matter. The liver, mesenteric glands, and all the other viscera of the abdomen, were natural: so, also, were those of the thorax. In short, the disorganization of the stomach constituted the only deviation from the healthy structure." 320.

*Case* 4. A female infant which had been freely purged of the meconium, and which appeared to be doing well, was seized on the 13th day from its birth with a smart feverishness, that lasted 24 hours, attended with intense heat of surface. There was no cough, vomiting, or diarrhœa; but a dark green matter was discharged from the bowels. The fever gone, the child remained fretful—had a tumid abdomen—drank often of gruel, but refused the breast—lost strength rapidly, and died on the 4th day of its illness. "*Repeated doses of scammony were given*," and the warm bath was occasionally used. It was attended by Dr. Thatcher, who furnished the particulars of the case.

*Dissection*. There was matter in the left cavity of the chest; but the lungs on both sides were sound. Much flatus in the intestines, but scarcely any solid or liquid matter. The stomach was completely distended with fluid. "At its splenic extremity, near to the cardia, and on its posterior surface, a part of it appeared almost pellucid, as if the inner coats were dissolved." By everting the stomach this was found to be the case, as the peritoneal covering only remained, and some few fibres of the muscular. Two other smaller erosions were found a little farther from the cardia. "*All round these erosions the villous coat appeared loose and detached from the other coats, and had a slightly reddish tinge.*"

These four cases are all that our author has to produce from his own observation, but on a little research he found a great number of instances recorded by others. These he has reduced to a tabular form, and they may be perused with advantage in the volume of Transactions under review. We must necessarily pass them over.

In respect to the theory of the disease, it seems evident to Dr. Gairdner that the erosions and perforations described above, do not arise from ulceration, "for they are found either without any indications of vascular action, or with only some slight redness of the villous coat." This does not appear quite so evident to us. Neither do we conclude with our author that wherever there is an aperture in the stomach, there must necessarily be extravasation of its contents into the cavity of the abdomen. The fact is, that every part of the stomach is at all times close contact with some other viscus, and, therefore, extravasation may not take place, although there exist a considerable aperture. Our author adopts the Hunterian doctrine, that these perforations are attributable to the solvent action of the liquids of the alimentary canal after death. "The presence of such appearances, in some cases, and their absence in others, may depend on variations in the quantity and quality of the contained fluids, and also on peculiar states of the containing parts, which render them of more or less easy solubility." It is the peculiar *softening* then of the coats of the stomach and bowels, occurring in infants, that constitutes the disease in question, according to our author. The following are Dr. Gairdner's therapeutical indications.

"Notwithstanding the absence of every appearance of vascularity of the stomach in many instances of this disease, it is evident that some degree of vascular action must attend its commencement, since it is hardly possible to account for the effusion of fluids between the coats of the stomach and intestines by any other hypothesis. I would therefore propose, if the child is tolerably stout, to abstract blood in this stage, and I should be disposed to apply a blister over the epigastrium, with a view to abate the local determination towards the vessels of the stomach. Both these remedies should evidently be applied *as early* as possible. Cruveilhier employed blisters, but applied them to the inside of the thighs, and they do not appear to have done any good. There are two other remedies, from both of which I have seen some benefit, or at least some temporary alleviation. I mean opiate enemata, and the warm bath. The former tend to check the diarrhœa, and they have both considerable influence in diminishing irritation. I should be disposed to avoid throwing into the stomach any food or drink calculated to irritate, either by its nature or by its quantity; and I should particularly avoid medicines of almost every description, but especially those of a purgative nature." 353.

It is evident, we think, that Dr. Gairdner has thrown little light on the nature of the perforation of the coats of the stomach. He contends, indeed, that it is *not* owing to inflammation or ulceration; but, we think, not very successfully. For our own parts, we are still disposed to attribute it to an inflammatory origin.

In an appendix, Number 2, a case is communicated to our author by Professor Alison, but contains nothing extraordinary.

---

18. *Gastritis—or Softening, Extenuation, and Destruction of the Mucous Membrane of the Stomach.* M. Louis, of Paris, has lately published a paper on this subject, in the *Archives Generales* for May—much more minute and refined, we fear, than useful or practical. Some instruction, however, is to be gleaned from it, and this we shall convey in as few words as possible. Twelve cases are detailed as the basis of the author's inferences and reflections. We shall notice the *post-mortem* appearances before the history or symptoms.

1. *Autopsic Phenomena.* The stomachs presented nothing particular externally. Internally there were sometimes patches of a pale white, but much more frequently of a blue colour. The mucous membrane, in the points corresponding with these appearances, was very thin and soft, transformed, as it were, into a glairy mucus. These spots were sometimes rounded and continuous—sometimes disposed in long and narrow bands. A slight examination would have led to the idea that, in these places, the mucous coat was entirely destroyed—and frequently there was actual destruction, but to a very small extent, of the membrane. Various degrees of this lesion, from slight extenuation to entire destruction of the mucous coat, were sometimes observed in the same stomach—exhibiting the progress of spontaneous perforation. These phenomena were sometimes detected in the lining membrane of the œsophagus, at its lower portion.

2. *Symptoms.* In most of the individuals there had been derangement of the digestive organs for a considerable period previously. The digestion had been disordered—and, in many cases, there had been wasting of the flesh, and loss of appetite. In some, there were pains in epigastrio, acidities, &c. The *immediate* symptoms were great diminution of appetite—gastralgia—shivering, followed by heat, thirst, and after a period, varying from a few days to a month or two, nausea and vomiting. These symptoms remained till death, sometimes permanent, most commonly intermittent. The only *invariable* symptom was nausea—in nine out of the twelve there was vomiting—in eleven out of twelve there was epigastric pain. The appearances of the tongue were various and uncertain. In four out of the twelve patients, it was natural. *Diarrhœa* existed in the majority of the cases—and also *phthisis*. Its progress varied from a few weeks to many months. The diagnosis can only, if at all, be gathered from the symptomatology. The *nature* of the disease appears to be inflammatory—sometimes acute, sometimes chronic. In respect to *prognosis*, little can be said—as in no case was the disease seen uncomplicated—in the majority of instances it was secondary of a mortal affection, *phthisis*, for example. Its causes are totally unknown.

We have thus given a short account of a paper which has occupied many pages of our foreign and domestic cotemporaries. As we said in the outset, we do not think it likely to contribute much towards the advancement either of pathology or therapeutics. It bears, however, on the subject of Dr. Gairdner's preceding paper.

---

9. *On some Diseases of the Stomach.* By M. Bourdon.*—These cases occurred principally under the eye and in the wards of professor Fouquier, and appear to be narrated with great fidelity and freedom from

---

* Recherches sur quelques maladies de l'estomac, observées à la clinique de M. Le Professeur Fouquier. Par M. Isid. Bourdon. [*Revue Medicale, Mai,* 1824.]

bias:—they are therefore valuable facts, and deserving of record. We shall, however, considerably abridge, as is our usual custom, the original documents, while nothing of importance shall be passed over in silence.

*Case* 1. A man (Lesage) 71 years of age, a shoemaker by trade, till the age of 53, when he lost one of his arms, and then changed his employment to that of a porter. He asserted that he had enjoyed good health till the year previous to his application. At that period, a swelling in the epigastrium, which had not before caused any inconvenience, became painful and increased in size, with loss of appetite, difficult digestion, and nausea. Finally, vomitings came on, especially after eating any solid food. He changed his diet to liquids, and the vomitings ceased for some months, when they returned, accompanied by colicky pains and constipation of the bowels. A fall at this period aggravated all the symptoms, and he was obliged to seek refuge in La Charité, August 1819. He was now meagre, yellow, his countenance indicative of great suffering. He vomited always about an hour after taking food. The epigastric tumour was large, and had augmented rapidly, according to the patient's account, since the constipation came on. A diarrhœa supervened after using some lavements, and the tumour lessened in size till it was no larger than an egg. It was irregularly rounded, situated to the right of the xiphoid cartilage, and two inches below it—very moveable in all directions, but particularly in a line from left to right—pulsatile in accordance with the action of the heart and arteries. It was considered to be an aneurism of the aorta or cœliac artery. The pulsations continued in it, whatever position was assumed by the patient, or to whatever side the tumour was pushed by the hand. The pulsation was occasionally perceptible to the eye as well as to the touch.

The nausea and pains continued, especially after taking food or drink—with progressive emaciation, and gradual abolition of all the senses, except that of sight, which remained unimpaired. At length dropsical effusions into the cellular membrane of the lower extremities took place—then in the trunk, with difficult respiration. The abdomen became swelled, painful, and sonorous at the epigastrium, with fluctuation in the hypogastrium. Fever came on, and death terminated his sufferings on the 25th August.

*Dissection.* Passing over some minor details, &c. to the abdomen. The peritoneum was inflamed and covered with false membranes in some places. It contained a yellowish effusion. The pyloric orifice of the stomach presented a rounded swelling, which did not extend to any other portion of that organ, nor to the duodenum. The passage was contracted, but would still admit the point of the little finger. The tumour was of a scirrhous nature. The pancreas underneath the tumour was sound; but the neighbouring lymphatic glands participated in the diseased state of the pylorus, and pushed that part forwards. In the centre of this scirrhous mass (beneath the pylorus) was found running a large artery, which proved to be the superior mesenteric, near its origin.

It was this, no doubt, which caused the pulsation in the tumour. The thoracic duct was pressed upon by the tumour and narrowed. This circumstance tended no doubt to accelerate the emaciation.

*Case* 2. A man, 35 years of age, had been a soldier for a considerable time, and then took to the trade of a shoemaker. In early life he had been subject to nasal hemorrhages, which were stopped by the habit of taking snuff. Later in life he became affected with a quartan ague, which lasted twenty-three months, and was ultimately cured by tonics. From this period he suffered from indigestion and occasional diarrhœa. The disease, which brought him to the hospital had commenced two years previously. It was a sense of constriction at the epigastrium, which was soothed by taking food. This constrictión was accompanied by sensations of dragging in the region of the stomach, a great afflux of saliva to the mouth—nausea—painful and tedious digestion—and latterly vomitings, with constipation of the bowels—emaciation—and pyloric jaundice, (l'ictere pylorique.) The vomiting did not come on till a considerable time after eating. Two or three times he brought up a quantity of half coagulated blood. He died four months after he entered the hospital.

*Dissection.* The emaciation was not so great as in cancerous affections of the stomach in general. The stomach was found adherent, towards the lesser curvature, to the liver, over a space of several inches. In the centre of this adhesion the stomach was ulcerated, the ulcer penetrating into the substance of the liver, and being surrounded with callous edges. Excepting where the ulcer was situated, the rest of the liver was sound. The pyloric orifice was contracted.

*Case* 3. Claude Dubois, a shoemaker, 29 years of age, of feeble constitution, bilious temperament, and a native of Paris, had also been a soldier, and had, in that capacity, contracted several times both syphilis and scabies, of which he had been badly cured. In August 1810, he got a fever, and an inflammatory bowel complaint, which last returned several times, and was accompanied by violent colicky pains and some pyrexia. Strong drastic purgatives were administered to him at this time, which produced copious evacuations upwards and downwards, followed by great emaciation, increase of the colick, and much debility. He was obliged to enter the hospital St. Antoine, where he was treated for misplaced or retrocedent gout, without success. He left that hospital and came to La Charité, in October of the same year. He was now jaundiced, emaciated, harrassed with pain in the abdomen, especially when he lay on his back. He had most ease when lying on the right side. His tongue was loaded, but he retained his appetite.—Digestion was slow and painful, particularly when he ate vegetables. He had the colicky pains often before eating. He never had any nausea or vomiting. He was constipated, but the constipation easily yielded at any time to gentle aperients. He experienced, in the evenings, flushings of heat, radiating from the epigastrium to the face. The right hypochondrium was sensible to pressure—the abdomen was a little swelled—the lower extremities œdematous—the urine abundant—the pulse always quick—a dry cough. He died in April 1810, never having experienced either nausea or vomiting to the last.

*Dissection.* A large fungous ulcer was found in the stomach, forming a band round its interior surface, at which place the coats of the organ were half an inch in thickness. The stomach adhered to the liver, but without ulceration at that part. It was also adherent to the pancreas, which was scirrhous. The liver was wasted. There was a scirrhous patch towards the termination of the jejunum—and some ulcerations of the mucous membrane, exhibiting no cancerous characters.

It is remarkable, that there should be no nausea or vomiting in such an extensive ulceration of the stomach. This is attributed by M. Bourdon to the sound state of the pylorus. He thinks also, that the universal adhesion of the stomach to the neighbouring viscera must have taken away the power of vomiting.

*Case* 4. A man, 40 years of age, who, for 39 years, had enjoyed good health, was affected with fever and inflammatory diarrhœa in the month of September 1807, which gave way to the usual remedies.—Two months afterwards, he felt, at the pit of the stomach, a weight and also lancinating pains, which were always worse towards evening and in the night. These symptoms went on increasing, till he came into La Charité in the month of February, 1808. He then complained of a painful lancinating heat in the epigastrium. No head-ache—no yellow tint in the complexion—respiration free and natural—but he was easily put out of breath by the least exercise. The pit of the stomach was sensible to pressure; but there was no perceptible tumour. He had no appetite—eructations after meals, the uneasiness remaining the same. He had neither constipation, vomiting, nor even nausea. The evacuations from the bowels were natural in all respects—but the digestion was slow, painful, and accompanied with much disengagement of air. He became greatly emaciated, and died rather suddenly under great pain, which opium had not the power to mitigate.

*Dissection.* The body of the stomach was sound. Its pyloric orifice alone was affected. There was here a small tumour round the orifice, the internal surface of which was ulcerated, which ulceration extended a little way on the mucous membrane of the duodenum.

Here, too, there was absence of nausea and vomiting, although the pylorus was ulcerated. The passage, however, into the duodenum was free.

*Case* 5. Galet, aged 62, a coal-porter, had always enjoyed good health and embonpoint. Even the day he entered the hospital, he was in good spirits, had a florid complexion, and exhibited all the attributes of a sanguine temperament. He had lately become affected with sharp pains in the epigastrium after the sudden healing of an old ulcer on the left leg. From that period he was troubled with acid eructations, and vomiting. He emaciated and got weak. He bartered his provisions for wine, which he took in excess. But this did not prevent the pains in the stomach, the vomiting, and the constipation, which was very obstinate. The physician considered the disease as gastritis, and the patient was twice bled at short intervals. The bleedings seemed to mitigate the pain; but two days after the last venesection, the patient died suddenly.

*Dissection.* Water was found in the cavity of the peritoneum—tubercles in the liver—a cancerous ulcer in the cardia of the stomach, the rest of the organ being sound.

This case goes to prove that no temperament is secure from cancer of the stomach—that scirrhus of the cardia brings on death more quickly than that of the pylorus, and without so much marasmus or sallowness of the complexion—that scirrhus of the stomach may be mistaken for gastritis—and finally, that venesection is rather a hazardous remedy in such a case.

A few observations are here introduced from the pen of Professor Fouquier, relative to the treatment of this melancholy class of human afflictions. It is needless to observe, that this can only be palliative.—The great objects are to mitigate pain and supply nourishment to the patient. The medicinal and dietetic substances which have been found most beneficial in Professor Fouquier's practice, were those which present a natural or artificial combination of light bitter or aromatic principle with a nutritive. Such is to be found in a decoction of iceland moss with milk—gruel and syrup of cinchona—light bitter infusions, &c. Calcined magnesia, and the carbonic acid have been occasionally found useful. Blisters to the epigastrium have sometimes suspended the vomitings. Glysters of broth and other nutritious substances are also to be had recourse to when the debility and emaciation are making progress.

*Case* 6. M. Delacour, a septuagenarian, of bilious temperament and melancholic disposition, had experienced for years past a sense of weight in the epigastrium, with irregularity of bowels, diarrhœa and constipation alternating. Two years previously to the date of report, he had dried up an old ulcer on the leg, since which the gastric affection increased—the appetite failed—the digestion was slow and painful—emaciation advanced—nausea but not vomiting obtained. On examination a small rounded tumour was felt a little below and to the right of the umbilicus. It was moveable, and painful on pressure. M. Fouquier pronounced its seat to be in the intestine or pylorus. The pain in the tumour was always most severe when diarrhœa was present. At all times, however, he felt as if a bar was across the hypochondria. Debility and emaciation now made rapid progress—the lower extremities became œdematous—colicky pains were excessive, and obliged him to use opium—the tumour enlarged in size—paralysis of one side took place a month before death closed the scene. He had no vomiting at any period of the complaint.

*Dissection.* There was a fungous ulceration embracing the interior of the right extremity of the stomach and including the pylorus. The exterior coats of the stomach, corresponding with this ulceration, were an inch in thickness. The left extremity of the organ was sound. There was an adhesion between the liver and the diseased portion of stomach, and the ulceration appeared to have penetrated to the substance of the former organ, which was scirrhous at that part. The colon also adhered to the scirrhous stomach, but its mucous membrane was not diseased, though somewhat phlogosed.

*Case* 7. A rope-maker, 48 years of age, had experienced some difficulty in swallowing for two years past. The food, in passing down, caused pain along the œsophagus extending to the stomach. During digestion the patient felt a sense of weight and distention in the stomach, with frequent acid or gazeous eructations. There were occasional vomitings—but no derangement in the function of the bowels. The appetite continued, though the digestion was slow and uneasy. The abdomen at length enlarged, and fluctuation became evident. It was also tender to pressure, especially towards the hypochondria. Emaciation made progress—the lower extremities became œdematous—and death at length closed the scene.

*Dissection.* The coats of the stomach were found scirrhous, and thickened throughout their whole extent. The interior also presented a crowd of vegetations or excrescences, about the size of peas. This diseased state did not extend beyond the cardiac or pyloric orifices.

Here it is evident, that vomiting was induced, not from the contraction of the stomach itself, but from the action of the abdominal muscles —a pathological fact that bears on the disputed physiology of vomiting in a very forcible manner.

*Case* 8. Cecilia Charpentier, 61 years of age, had enjoyed good health from infancy, and continued to menstruate till the 50th year. About twelve months before the date of report, she had erysipelas of the face, accompanied by fever and delirium. While convalescing from this complaint, she began to feel pain and sense of heat in the epigastrium. At night the pain was more violent, and became lancinating—often causing her to lie on her face in bed to procure some ease. The appetite now gave way—the embonpoint disappeared daily—the nights were passed without sleep—for pain and perspiration left her no intervals of ease. When examined at the hospital, the physicians felt, though obscurely, a tumour in the epigastrium, of uneven surface, and stretching towards the umbilicus. From this place the pains radiated to the spine and mammæ. The respiration was free, and no suspicion was entertained of any disease in the chest. Œdema now took place over the body, and even the face. There was no nausea or vomiting; but a considerable flow of saliva from the mouth. The pulse was frequent—water fluctuated in the abdomen—diarrhœa supervened, and death closed the scene.

*Dissection.* The right lung was hepatized throughout two thirds of its volume. When squeezed, a quantity of purulent-looking matter issued forth, as is usual in lung long hepatized. The liver descended low, and was adherent to the lesser curvature of the stomach. The tumour felt during life, was formed by the stomach and the glands in its neighbourhood. On the internal surface of the lesser curvature of the stomach, and near the pylorus, several fungous excrescences were found, leaving the pyloric orifice, however, free.

It was remarked in this woman, that *pressure* always mitigated the abdominal pain—"an almost infallible indication," says M. Bourdon, "of adhesion between the stomach and some neighbouring viscus, especially the liver." On the contrary, the pain was always increased by the *decubitus dorsalis*. The most remarkable phenomenon, however, in this case, according to M. Bourdon, was the fact of the hepatization of the lung escaping the notice of the physicians. We do not wonder at this circumstance, considering the severe abdominal disease, which was capable of masking a far greater extent of pulmonary affection than existed in this patient.

M. Bourdon makes some observations on, and draws some conclusions from the foregoing cases; but they are such as will readily occur to the mind of the practitioner, on perusing the documents which we have now laid before him.

---

10. *Abscess in the Parietes of the Stomach.** A curious case of this kind is related by Surgeon Callow. A man officiated as hospital cook in the 96th regiment, till the day of his admission, 34 hours prior to his death—no antecedent indisposition being complained of, except loss of appetite, sickness, and diminution of strength. When received into hospital, he had violent pain in epigastrio, incessant vomiting, quick pulse, cool skin, brown and dry tongue, constipated bowels. In a short time, the pain shifted to, and became fixed at the umbilicus, assuming the character of enteritis. In 24 hours, one hundred ounces of blood were abstracted, locally and generally; but the patient suddenly expired in a violent fit of vomiting, having passed a quantity of purulent matter by stool.

*Dissection.* Diffused inflammation over the small intestines. A quantity of bland pus floating among the viscera, and issuing from a lacerated aperture in the small curvature of the stomach, around which the coats of this viscus were in a state of disorganization. All the other viscera were sound. It is supposed by Mr. Callow, and with reason, that the pus must have been contained in a cyst. It amounted in the whole to more than seven pounds. While in the cyst, it appears to have excited little irritation; but the moment the cyst burst and the matter came in contact with the mucous surfaces, they were excited into a state of inflammation.

The dissection of the above case nearly cost Mr. Callow (an able and meritorious medical officer) his life. The thumb of the right hand was pricked by a spicula of bone, while being immersed in the purulent

* Surgeon Callow, 96th Regiment. Med. Journal, No. 306.

matter. At 12 o'clock of the next night, while on a journey to London, Mr. C. was attacked with acute pain in the thumb, and soon after a shivering fit. The journey was persevered in, although the pain invaded three of the other fingers, and violent rigors rapidly succeeded each other. Arrived at the end of his journey, he retired to a warm bed, and medical assistance was summoned. A deadly coldness prevailed for some hours, more especially in the lower extremities, which were nearly destitute of circulation. The hand swelled, and the pain extended up the fore-arm. A violent reaction succeeded the cold stage, and the sensations during this paroxysm were of a most insupportable kind—"bearing a stronger affinity to an attack of hydrophobia than to any other disease to which it could be assimilated." The head-ache was so violent "that the brain appeared on fire," while rapidly succeeding spasms of the diaphragm threatened to suspend respiration. Deglutition of fluids was exceedingly difficult, while thirst was excessive. Vivid red streaks in the course of the lymphatics indicated the progress of absorption. The warm bath produced much relief, while leeches to the arm diminished pain, for a time—then all the symptoms became exasperated. The same measures were again and again put in force, with temporary benefit. The axillary glands swelled. A paroxysm took place every twelve hours regularly, at which periods, there was much pain in the right side of the thorax, attended with difficult respiration. The progress of the absorption seemed stopped at the bend of the fore-arm. After the fourth day, there was great disposition to the formation of depôts of matter in the arm, but fortunately they did not take place. After a week, some amendment took place, though slowly. At the time of writing (five weeks from the accident) the functions were much deranged, and Mr. Callow has lost the use of his hand. We congratulate him on the preservation of life. Few have lived to tell the tale, after being in his condition.

---

11. *Cynanche Trachealis.** Dr. Meyers, of Manningtree, has lately published some cases of this formidable disease, accompanied by judicious observations on its pathology and treatment. He justly considers it as the result of inflammation of the lining membrane of the trachea, on which an extensive morbid secretion is thrown out, quickly assuming a thick gelatinous consistence, and consequently preventing the action of the air on the blood in the lungs. The first case related by Dr. Meyers proved fatal on the fifth day of the attack, and twelve hours after our author saw him. The disease therefore may be said to have had its own way, and the appearances on dissection, under such circumstances, are interesting.

"Upon laying open the trachea, from the cricoid cartilage to its termination in the bronchial tube, I found the whole cavity so completely

* Dr. Geo. Meyers. Med. and Phys. Journal, October, 1824.

blocked up with dense coagulable lymph, that I could with difficulty pass the handle of my knife along. I carefully at length separated a large portion of it, and was struck with the florid appearance of the internal coat of the trachea, much resembling scarlet cloth as to its colour. It will be observed, this disease had been of five days' standing only, and must indisputably prove the rapidity with which it advances, and the impracticableness of removing it when thus formed and fixed. This dissection likewise throws additional light upon the treatment necessary to be adopted and pursued; and, although it may not afford any reason for employing a fresh or novel plan, most unquestionably tells us that our efforts hitherto made must be more vigorously followed up." 209.

Our author's plan of treatment is judicious. Bleeding, local and general—blistering—purging, and the exhibition of mercury, are to be had recourse to, and put in force without loss of time. Emetics are highly useful in this disease, both as checking the inflammatory exudation, and loosening it when formed.

---

12. *Perforation of the Intestines.* In the ARCHIVES for September last, there is a case of this kind by Legallois, with comments on it by Bouillaud. The patient was a watch-maker, 23 years of age, admitted into the Bicêtre in January, 1823, for pains in the abdomen, which yielded, and he was discharged. In the beginning of May he returned, and an irregular tumour was detected in the abdomen, occupying apparently its whole extent, but more conspicuously the right iliac region. The pulse was frequent and contracted—skin dry and harsh—abdomen tense, like the parchment of a tambourine, but without meteorismus. Pressure gave pain. Antiphlogistics and counter-irritation gave no relief. He became harrassed with porraceous vomitings and a colliquative diarrhœa. Emaciation advanced—he passed substances resembling portions of the intestines themselves, and died, after fifteen days of suffering, in complete marasmus.

*Dissection.* When the peritoneal cavity was laid open, a quantity of matter, similar to that which had been passed by stool, issued forth. All the abdominal viscera were glued together. The omentum was very much thickened, and adhered to the circumference of the pelvic basin. The convolutions of the intestines were agglutinated, and seemed as if macerated in the liquid contained in the abdomen. The pelvis was a complete sink, in which were collected all the fæcal matters that had escaped into the peritoneal cavity from the cœcum and descending colon, both of which were full of perforations. The peritoneum was red throughout, and, in some places, approaching to gangrene. On this agglutinated mass was seen a multitude of small productions of a whitish caseous matter, varying from the size of hemp-seed to that of a large pea, evidently of a tubercular nature. The liver and stomach were natural in their internal structure. Throughout the large intestines there were many perforations—fifteen at least, the smallest of which were of the size of a two franc piece, and the largest more than two inches in diameter. These last chiefly occupied the cœcum and transverse colon. The latter portion of intestine was double its natural length, being several times folded on itself in the right iliac fossa. All these perforations were without adhesions. In some, the margins were smooth, and had a cicatrized appearance. There were several perforations in the small intestines, but these were closed by adhesions existing between their convolutions, and between them and the neighbouring organs.

M. Bouillaud, in his comments on the above case, mentions an instance which came under his own eye, in the Hospital Cochin, where the intestines were perforated like a sieve. The patient died of pulmonary consumption complicated with uteritis. If it be asked how life is preserved, for some time, under such circumstances? it may be answered that, effusion of the fæcal contents is not a necessary consequence of perforation of the intestinal tube. Nature often repairs with one hand the ravages she is committing with the other. It is sometimes so here. The adhesions formed, during the inflammation preceding the erosion, occasionally check the issue of fæcal matters out of the intestines. It sometimes happens also that openings are formed through the adherent portions of intestines, and thus false passages made from one convolution into another. Openings of this kind have taken place between the intestines and bladder—and even between them and the uterus. Such temporary checks to the fæcal effusions only occasionally take place. More frequently the intestinal contents get abroad into the general cavity, and the consequent peritonitis accelerates the fatal event.

---

13. *Suppression of Urine.** Suppression of the urinary secretion is, at all times, a most formidable disease. There are some peculiarities, however, in the following case, which render it remarkable.

Mr. Robinson, 41 years of age, corpulent and robust, of sober habits, and by trade a brazier, had been for three years subject to gout of an irregular kind. On the 4th April our author was called to him. He was just recovering from an attack, which had stopped suddenly on the 2d. He had now pain in the left iliac region—the tongue was clean—pulse regular. Castor oil and tincture of senna were given, and caused three or four bilious dejections, with complete relief. 6th. The pain in the iliac region returned—pulse 70, and full. Leeches to the pained part. One grain of opium, three of calomel, and ten of extract of colocynth. A turpentine glyster in the evening. 7th. Informed our author that he had passed no water since the morning of the 5th. Is now free from pain. Pills of calomel and aloes opened the bowels freely. The catheter was introduced, but no urine came away. 8th. He slept during the night, and said he felt quite well this morning, were it not for the stoppage of water. Tongue clean—pulse 70—no head-ache. Twelve ounces of blood to be taken from the arm. *Evening.* Blood

* Dr. Teeling. Dublin Transactions, vol iv.

buffed—had four liquid stools—no urine. Warm bath—blister to the region of the kidneys—a diuretic mixture. 9th. Dr. Percival in consultation. No urine. Slept in the night. Fourteen ounces of blood to be drawn from the arm—the bath to be repeated—two grains of calomel and two of camphor to be taken every two hours, with a saline diuretic draught. 10th. Four stools yesterday—no urine, except five or six drops—slept some in the night. Has taken two drops of the oil of cageput every four hours in addition to the other medicines. The same to be continued. 11th. Took an aperient last evening, which operated well. A few drops of urine passed three or four times, amounting in the whole to about half a drachm—pulse 80, soft and regular. The medicines to be continued. *Evening*. Had smart gouty pain in both knees for four hours—then suddenly ceased. 12th. No material alteration. The gums are affected by the mercury—some flying pains in the left hypochondrium. *Evening*. Passed about 24 drachms of pale yellow urine at four efforts. Six leeches to the region of the left kidney. 13th. Slept eight hours—passed a little urine with his stools. Passing on to the 16th, Dr. Tuomy met them in consultation. The patient had had little sleep in the night—was disposed to be incoherent this morning—pulse 80—hiccup—great anxiety—abdomen appears somewhat tympanitic—no urine. 17th. Stools passed involuntarily—no urine—convulsions—extinction of voice. 18th. Died comatose.

" *Dissection*. The omentum was diseased, and both kidneys were surrounded by an immense quantity of fat. The right kidney was diminished in size: the cavity of its pelvis was filled with calculi, of a whitish-grey colour, and rough surface, many of them as large as small peas. The whole internal or secreting surface of the kidney was firmly coated over with a fine kind of gravel, resembling pulverized free-stone; and in the upper part of the ureter belonging to it a calculus was found, as large as a small almond, which completely blocked up the passage. No urine whatever appeared to have been secreted in this kidney.

" The exterior of the left kidney was of a purple-red colour. In its pelvis there was a small quantity of urine, together with a few calculi similar to those found in the right kidney, but there was none of the coating gravelly matter. The bladder contained a few calculi, and some urine. The other abdominal viscera were perfectly healthy." 180.

It is evident from the above case and dissection, that it was the cessation of *function* in the kidneys, rather than any alteration of structure, which caused the death of the patient.

---

14. *Hepatic Phthisis*.* That hepatic disease often simulates, and what is worse, often produces disease in the lungs, must be known experimentally by every attentive practitioner. But the difficulty of ascer-

* Sir T. Moriarty. Irish Transactions, vol. iv.

taining when real disease of the pulmonary structure has actually commenced, can only be known to those who watch with zeal and assiduity the phenomena of disease in the living body, and mark well the ravages which it leaves in the organized structures after death.

The subject of the present paper was a married lady, about 35 years of age, who, for some years, had been afflicted with severe pains in the stomach, for which aromatic tinctures, æther, and even opium were had recourse to. She was of temperate habits, apparently scrofulous, and had borne children. At length a teazing cough came on, with dyspnœa and occasional hæmoptysis, followed by evening fever and night sweats. Previously to our author's attendance, she had had head-ache and hemiplegia, but from these she was entirely recovered. She was considered as phthisical, and ordered milk diet and country air. This was in 1804, when Sir T. M. first saw her. On examination he was struck with the absence of any emaciation or acceleration of pulse. He found nearly the entire of the convex surface of the liver indurated and uneven to the touch. "Both lobes were evidently diseased." She complained of acute pain in the right acromion and between the scapulæ—tongue furred—bowels slow—catamenia regular. For the present she was advised to remain in the country— to regulate the bowels with senna and tartarised soda, and to use camomile tea acidulated with sulphuric acid. After some months she returned to town (Roscommon) much improved in appearance. Still she had recurrences of the hæmoptysis; but as this was considered as merely symptomatic, half a drachm of mercurial ointment was ordered to be rubbed in every night on the right side. When about an ounce of the ointment had been used a smart mercurial fever prevented its further exhibition, the gums being slightly affected. She soon recovered from this; but a considerable period elapsed before the remedy could be resumed, (for what reason is unknown). The interval had been spent in Dublin, under the care of Dr. Plunkett, who tried the nitric acid and saline bitters, without any advantage. After a period of seven months the head-aches returned—the vessels of the eye became turgid—the pupils permanently contracted on the application of light—with sense of swimming and confusion in the head. These symptoms were soon followed by another attack of hemiplegia, which soon gave way to leeches, blisters, &c. At the solicitation of the patient, Sir T. M. was induced again to try mercury, after an interval of twelve months from its former exhibition. A recurrence of febrile irritation and sharp pain in the side again suspended the use of mercury. The side was relieved by leeching. During the two subsequent years the patient had frequent hæmorrhages from the lungs. One night, at midnight, our author was called up to witness the following phenomena:—

"The mouth gaped open, the lower jaw hung, as if dislocated, on the sternum, with such sudden agitations of it against the upper one, as imparted a tremulous motion to the whole body and bed. This gaping posture sometimes continued for minutes, and presented to the eye all the appearance of dislocation."

"Liniments with laudanum, æther, camphorated spirits of wine, and turpentine, were tried without any advantage. It at length occurred to me that pressure on that twig of the third branch of the fifth pair of nerves, which passes through the foramen at the chin, might be beneficial: the experiment succeeded, the oscitation having immediately ceased. On removal of pressure it again returned, but was stopped by permanent pressure; a silver coin was kept on by a bandage.

"This vibrating oscitation returned frequently at subsequent periods, but was always removed by the same means, which the patient herself was now in the habit of applying." 19.

Our author now determined to adopt a merely palliative treatment. The side was generally relieved, for a time, by leeches, and the head by blisters. At length she was attacked with severe paroxysms resembling asthma, from which she was always relieved by assafœtida glysters and tincture of senna. These usually brought away much bilious and slimy fæces. The liver still continuing apparently to be diseased, mercury was a third time resorted to, and with the same effects as before. The physician now resolved to leave the complaint to nature, merely keeping the bowels soluble. "During the following year she had frequent shiverings; the induration of the side felt softer and more elastic, and suppuration appeared evidently to have taken place. She was at length seized with symptoms of dysentery; the side became more flaccid and shrunk: the tormina and tenesmus were excruciating. Frequent enemas, and purgatives of calomel and cathartic extract, brought off incredible quantities of fetid, sanious, and purulent matter; quantities of pus were also suspended in the urine, which produced much irritation in its passage through the urethra. In some weeks the dysenteric symptoms abated in violence; but for a period of more than a year, evacuations of purulent matter, and with it quantities of a gritty substance, like coarse sand, continued to pass by stool; any delay of it in the rectum excited torture, and required constant enemas and brisk purgatives for its removal.

"The pulse, which hitherto shewed little variation, now lost its intermission; the primary, as well as secondary symptoms of her disease, gradually disappeared; but extreme debility succeeded, which neither tonics, generous diet, or change of air, could prevent from increasing. The blood appeared deoxygenated, and the capillary vessels to have lost their tone. The surface of the body became covered with large purple ecchymoses; blood flowed from the uterus, the rectum, the lips, the nose and the skin, and the legs swelled; to these bandages were applied; the mineral acids were given abundantly in water, or infusion of roses or cinchona, as the state of the stomach admitted, and claret was given in a reasonable proportion. Under this plan she rallied surprisingly, and was enabled to take exercise in a carriage; this was in 1813. After some months of gradual recovery, she persuaded her husband to reside in Dublin, where, after a short residence, she expired." 21.

The above is certainly a remarkable case. There can be little doubt, we think, that both the cerebral and thoracic symptoms were sympthetic of the diseased liver; but we consider it quite a misnomer to call the disease hepatic phthisis. Hepatic *hæmoptysis* it might have been at one time; but assuredly there were no evidences, that we can see, of *phthisis* in any stage of the complaint. One remark more, and we have done. Where the liver is so disorganized as to be felt indurated and knotty by the hand, it is madness to exhibit mercury, with the view of salivation. Local depletion and continued counter-irritation, with gentle aperients, and slight alterative doses of mercury, will do far more than salivation.

---

15. *On Anomalous Gout.* By A. L. G. Bayle.* Few diseases are involved in more obscurity than gout, whether we look to its nature, or its treatment. Few diseases veil themselves in more disguises than this proteian malady. The multiplicity of its symptoms, the complexity of its forms, and the obstinacy of its course were such as to attract the attention of physicians in all ages, since the days of Hippocrates. But they have all failed in ascertaining its proximate cause—and what is of more consequence, its cure. M. Bayle, has, in the paper under consideration, brought forward some curious examples of the numerous metamorphoses which gout undergoes, and which shew the necessity of being acquainted with the masks which it frequently assumes, as well to avoid errors of diagnosis, as to apply a rational method of treatment.

Case 1. Madame G——, 40 years of age, of a nervous temperament and delicate constitution, had enjoyed good health till the age of seventeen. Her grandmother had been a martyr to gout. In the course of her first pregnancy, and when only in her seventeenth year, she had an attack of strong convulsions in the muscles of her limbs and face, which lasted about half an hour. Ten years afterwards she had a similar attack in her second pregnancy, the day after being bled. During the last five or six years, domestic afflictions have rendered these paroxysms much more frequent. She became affected with deafness at the age of 24, which has continued ever since; and she is also subject to pains in her teeth. At the age of 30, she began to have dull pains in the joints of the feet, with hard indolent swellings resembling nodosities. The same, in some degree, in one of her hands. From 1818 till the beginning of 1823, Madame G—— had often felt darting pains in the hands and feet, which did not, however, prevent her from going about her usual occupations. In the month of May, 1823, she was seized, some days after suppression of the menses, with intense hemicrania, accompanied by heat of skin and fever, and for which leeches, aperients, antispasmodics and sinapisms were used. Next day there was some mitigation of the hemicrania, while the feet swelled, became red, and painful. In a few days, both the hemicrania and the affection of the feet went off entirely. In August of the same year, she had a repetition of the hemicrania—and

* Revue Medicale, Juin, 1824.

since that period two other accessions, all which were cured in four or five days by the means above-mentioned. About 14 years ago, our patient, after some mental vexation, became affected with a dry cough, which was suddenly followed with complete extinction of voice, there being no pain, nor inflammation of the larynx. All sorts of remedies were tried in vain for the removal of this complaint. At length she remembered that her mother was cured of a similar affection by swallowing an ice. Madame G. had recourse to the same remedy, and with the same success as her mother. Since that period, she has usually lost her voice three or four times a year (generally after some chagrin) and always recovers it by swallowing ices. In the month of May, 1824, when the report closes, this lady was in the enjoyment of perfect health.

M. Bayle makes several reflections on the above case, which we need not introduce here. On a superficial view of the case, it will appear to many to present a succession of purely nervous affections unconnected with each other; but, on mature consideration, we are disposed to think, with our author, that they all, or most of them, had a connecting link, and that link gout.

*Case* 2. J. B. A——, 33 years of age, of nervous temperament, had been rickety in his infancy, but by the time he arrived at the age of puberty he enjoyed good health. He was even robust, and fresh-coloured. A deformity in the chest, however, still continued, the right side being much more prominent than the left. His mother had been delicate and nervous—his father gouty—his sister has been deaf for many years, and subject to convulsions, whenever she experiences any mental agitation.

Mr. A. had scarcely attained puberty when he gave himself up to venereal excesses—and so early as the age of 20, he experienced dull and uncomfortable pains in the joints of his feet, accompanied by slight swellings of the same. These went off in about a fortnight, and now commenced a profuse discharge of perspiration from the feet, especially during the night. From this time till the age of 30, he rarely passed a year without a fit of sickness of some kind. He had occasionally also pains and redness in the joints of the feet: and in the years that these occurred, he was in much better health than when he missed the visitations of gout. In the latter intervals, he was subject to numerous and strange affections—sometimes to the most alarming faintings—sometimes to apoplectiform seizures, during which he lost sense and motion, the pulse and respiration remaining free—at others, to such painful fits of cardialgia as caused him to cry out—ending in vomitings of porraceous matters which often lasted 24 hours without interruption, followed by a quick restoration to health. Finally, his illnesses sometimes assumed the form of copious and painless diarrhœa, lasting ten, fifteen, and even thirty days. During each and every of these attacks the urine and perspiration were very fetid.

In his 31st year, being the year 1821, Mr. A. enjoyed good health, with the exception of a sense of weight in his head, some dyspepsy, and a disposition to sleep after dinner. In May of the same year, he had demonstrations of gout in the feet for several days. On the 7th of the following month (June) he suddenly became senseless, his face being pale, pulse scarcely perceptible, extremities cold. He was bled, and sinapisms were applied to the knees.* He soon recovered from this state, and for some days afterwards he had copious vomitings of many coloured liquids, with quick pulse, heat of skin, head-ache, and alarming attacks of syncope. In about two months he was restored to his usual state of health. In the autumn of the same year, he made a journey into the country, during cold weather, and was seized, some days afterwards, with acute pains in the epigastrium, and greenish vomitings, which lasted eight days. After this he was troubled with great noise in his ears, which augmented a deafness to which he had been for some time subject. In the spring of 1822, he had wandering and lancinating pains in his feet, followed by dry cough, and in a few days, hæmorrhage from the lungs of about six ounces in quantity, with pyrexia. *Blood-letting, leeches, sinapisms to the lower extremities.* In eight days he recovered from this attack. Between this period and August 1823, he had two slight attacks of gout in the feet. In the beginning of September, during cold weather, he was seized with dry cough, which continued till the middle of October, when he one night brought up ten ounces of blood which quickly coagulated in the basin. Immediately after this he felt himself quite well. Two days afterwards, a fresh attack of hæmoptysis, with dry cough and pyrexia. He was bled to eight ounces, which produced syncope of five minutes duration, and great alarm lest he should not revive. On recovering, he was better, but the cough continued throughout the month of November, in unconquerable paroxysms, and with acute pain in the region of the diaphragm. His breathing was now habitually tight—he had irregular accessions of fever, especially in the evenings—sometimes anorexia, sometimes appetite with painful digestion, nausea, and cardialgia when he indulged in food.—These symptoms continued, with exacerbations and remissions, till the end of the month. The remissions were accompanied or preceded by depositions in the urine, or fetid perspirations of the feet. Towards the end of November, he expectorated yellow and thick sputa tinged with blood—he was affected with dyspnœa—irregular diarrhœa, progressive emaciation, acute pain under the three false ribs of the *left side*, with swelling and redness of the skin in that quarter, the other symptoms before enumerated continuing the same. Towards the end of December, there occurred two or three accessions of most alarming syncope. Early in January, though reduced almost to a skeleton, he had an attack of gout in the feet, which were red, swelled, and extremely painful. During this accession the dyspnœa disappeared, but the other symptoms continued. On the 11th January, death put a period to his sufferings.

* This was surely bad practice—we mean the bleeding, under such circumstances. That he did not die under the operation of blood-letting, was a god-send of good luck to the practitioner. *Rev.*

The same remark which was made on the first case, is applicable to this—namely, that, however various and apparently dissimilar this unhappy man's ailments, they were all referrible to a gouty diathesis. Every man in practice, who keeps an attentive eye on the phenomena of diseases, will see examples more or less nearly approaching to those which we have stated. The elder Parry has related many cases bearing great analogy to these, in his admirable, but unfinished "Elements of Pathology."

*Case* 3. M. A. 46 years of age, a Comptroller of the Customs, of full habit and sanguine temperament, contracted debts during a protracted intermittent fever, which he was not afterwards able to discharge. Other misfortunes followed, which produced a melancholy state of mind. He was sober, active, and temperate in his food. In the month of January, he had an attack of gout, which confined him a fortnight to the house. Greatly embarrassed with debts, and labouring under much anxiety of mind, he came to Paris, on the 29th December, 1820. His nights were now passed without sleep, and in a state of agitation, experiencing, at the same time, inexpressible pain in the head, and violent palpitation of the heart. Every thing presented itself to his imagination in the most gloomy colours. These symptoms increasing, he was, on the 2d Jan. in a state of actual derangement. Seized also with the desire of suicide, he distributed what little money he had to those who were nearest to him, and hastened out of Paris, without knowing whither he went, carrying with him a knife, with which he inflicted several wounds on himself, but none of them mortal, the knife being very blunt. He next resolved to drown himself; and arriving at the Pont de Neuilly, he was on the point of precipitating himself into the river, when he was prevented by a female, who, catching hold of him, exclaimed, "*what will become of your soul?*" He went into a Cabaret, where he passed the night. Next morning he attempted to hang himself, and nearly succeeded. On recovering from a state of insensibility into which he was thrown, he found himself quite bereft of sight; but this gradually returned, together with a glimmering of reason, which induced him to resist the desire of suicide. But this he could not long do. On returning to Charenton, he endeavoured to drown himself in the Marne, but was prevented by the thickness of the ice. Here he was secured and conducted to the Royal Asylum at Charenton, as a maniac. On examination, he was found in a high state of maniacal excitement, and was twice bled with benefit. Nevertheless, his face continued red, his pulse full and hard, his heart violently palpitating, and his mind in a state of profound melancholy. He remained at Charenton in a state of mental derangement, with much excitement, till the 7th January, when a fit of gout attacked both feet, and presently he was in a state of sound mind. He could not, however, perfectly recollect all that had passed during his phrenzied condition. The gout ran a regular and pretty severe course, and the mental alienation returned no more.

The reader must here determine in his own mind whether the gout or the moral afflictions were the cause of the mental alienation. For our own parts, we think the melancholy moral emotions prevented the regular course of gout to the extremities, and threw it on the sensorium, with the consequent tendency to suicide and insanity. This idea is strengthened by the evident fact, that a regular attack of gout in the feet instantaneously dissipated the maniacal hallucination.

We shall introduce one more case, and then bring this paper to a conclusion.

Case 4. Madam Eliz. D——, 40 years of age, of nervous temperament, had had, at different times, several attacks of regular gout in the feet, and sometimes in the upper extremities. Occasionally, however, it took an anomalous form, and attacked the stomach with violent pains, vomitings, and severe colick, which did not cease till gout came to the extremities. Some pecuniary losses now occasioned a slight and temporary derangement of mind. Afterwards the assassination of her husband deprived her completely of reason for a time, which alienation has since returned often. In 1812, she threw herself into the water, and nearly drowned herself. She was then confined at Rouen, each time that the maniacal hallucination returned. She was set at liberty in the intervals. Two years ago, she became affected with violent cephalalgia, accompanied by frequent and painful vomitings, especially after taking any aliment. An accession of mania dissipated all these symptoms. Soon after the menses became suppressed, followed by menorrhagia and frequent syncopes. One year ago, an accession of mania was preceded by most severe gastric affections, faintness, and hypothymia.

On the 28th August, 1821, she entered the Asylum at Charenton, presenting the following symptoms, viz:—redness of the tongue, vomitings, somnolency, faintings, cephalalgia. These symptoms ceased, and an accession of mania came on, with constant agitation, running about, crying, singing, loquacity, and great inclination to mischief. The features were altered, the face pale, with the appearance of some abdominal affection. In two or three weeks these symptoms diminished, and she had frequent alternations of tranquillity and agitation. By the month of February, 1822, reason was completely restored. At this time, the tongue became red, with epigastralgia, vomitings, cephalalgia. The vomitings continued some time, but the pains in the head and stomach went off soon. Towards the end of April, the gastric symptoms returned, with anorexia, pasty mouth, great tenderness at the pit of the stomach, with frequent bilious vomitings, provoked by the smallest quantity of aliment. It was only now that the physicians became acquainted with the patient's true history, and learnt that she had been subject to gout. Large sinapisms were therefore applied to the feet and the legs. Next day there was considerable amelioration of the symptoms, one of the feet being red and inflamed with gout. The day following, all the symptoms except those of gout had vanished. After this period, however, there were several alternations of gout and mania, the one never being present at the same time with the other.

From these and other cases detailed in the work before us, M. Bayle has come to the following conclusions:—

1mo. That the *nature* of gout is as yet unknown, and consequently that none of the theories broached in explanation of this disease, are at all satisfactory. It is neither a pure inflammation, nor an organic lesion—but apparently the result of a morbid change in some of the humours that circulate through the animal economy.

2ndo. That gout is capable of affecting any or all of the organs and tissues in the human frame, although it appears to have a greater predilection for some than for others.

3tio. That the symptoms by which gout is manifested, are those of inflammation—of the neuroses—of the hæmorrhagiæ—exhibiting the greatest variety of phenomena, which may exist separately—successively—or alternately, according to the intensity of the malady, its regularity or irregularity, the strength of predisposition, and various other circumstances by which the patient is surrounded. Hence the diagnosis of gout, in its anomalous forms, is often extremely difficult.

4to. That, notwithstanding the boasted assertions which are every day made, there exists no specific remedy for gout. At the same time it must be acknowledged that there are many medicines which are very useful in combating particular symptoms and forms of the disease, though there is none that strikes at the root of the evil—the vitiation of the fluids, its probable cause. There is but one indication which is universally applicable to gout—and that is, to cause a revulsion to the extremities by all possible means, whenever it affects an internal organ or tissue.

---

16. *Acute Cerebral Affections.** George ——, ætat. 17, a servant, of a lymphatico-sanguineous temperament, was attacked in the autumn of 1823, with a fever, which presented different types, and ceased and reappeared several times. Towards the middle of December, he complained only of pains in his legs, with, at times, paroxysms of fever, not however such as to confine him to bed. His parents, with whom he was, applied a blister to the inferior-anterior part of the fore-arm, in the direction of the cubito-radial muscle, with temporary advantage to the patient. But on the 10th and 11th of January, 1824, the fever returned, without any alarming symptoms. On the morning of the 12th, when he arose, his understanding seemed much disturbed; he breakfasted and dined heartily, but still his ideas and conversation were incoherent and confused. About eight o'clock, p. m., being seated at the fire, with his father and mother, he suddenly grew weak and fell senseless to the ground: he was carried to bed speechless, and M. Vial being called in, found him in the following condition. He lay supine; the eye-lids wide open discovered the pupil much dilated, and the conjunctiva slightly injected and reddened: no impression on the retina from a

---

* Gazette de Santé. No. xxiv. Aout, 1824.

lighted candle: body insensible to pinching; the arms and legs contracted and half bent: the upper and lower jaw, contracted also, with difficulty admitted some spoonfuls of liquid: hearing apparently quite gone; and his parents said that since the accident (about four hours previous) he had not uttered a single word: sense of smell unaffected by the odours of æther and liquid ammonia; respiration was pretty natural, and the pulse somewhat quick, but regular. As he had eaten pretty heartily, three grains of emetic tartar, in four spoonfuls of luke-warm water, were given, but with no effect. Two blisters were then applied to the legs and sinapisms to the soles of the feet, the latter to be renewed to the knees, four hours afterwards; eight leeches were ordered to the temples, but five only were employed, which produced very little bleeding. Diluents were given, and an ethereal draught taken every hour.

On the 13th, the patient was much the same; during the night and morning he had, at intervals, cried faintly: the urine was passed involuntarily. (Drinks as before, and two lavements, with an ounce of sulphate of soda in each).

The patient continued thus till the 16th, when he recovered his speech, but was delirious in his replies: the retina had become sensible to light; he had at times frightful dreams, at other moments he was quite tranquil; hearing again natural; the contraction of the limbs and jaws had ceased; the tongue was slightly red at the tip, and furred in the middle; the urine deposited, on cooling, a lateritious sediment, and was no longer passed unconsciously: there had been two stools in the twenty-four hours; the skin was a little moist. As the young man complained of his head, three leeches were put on each temple, and sinapisms on the inside of each thigh.

On the 17th, the sixth day of his illness, the delirium had greatly abated since the application of the leeches, the bites of which had bled freely. He continued to improve rapidly from this day, and on the 20th, he was very well and soon able to resume his ordinary occupations.

M. Vial, the author of the above statement, considers the case as one of inflammation of the cerebral substance—*encephalitis.* But Dr. Martinet, who was ordered to report on the case, by the Athénée de Medecine, justly looks upon it as an affection of the membranes only. A disease of the encephalon itself continuing so many days, with little amelioration, would, in all probability, have produced such organic lesion in the soft cerebral mass as would have ended in death, or induced a train of symptoms that would not have been speedily removed. It is therefore, we think with M. Martinet, to be placed in the class of *meningeal,* rather than *cerebral* inflammations.

---

17. *Tetanus.* Mr. Carmichael has published six cases of tetanus in the fourth volume of the Dublin Transactions, with the results. Of these, only one case terminated favourably. This was a man, 24 years of age, admitted into hospital on the 3d August, 1822, with pain and stiffness of jaws, difficulty of swallowing, risus sardonicus, pain at the pit of the stomach, confined bowels, pulse 90, and strong. The patient had received a contusion on the right shin three weeks previously. The tetanic symptoms commenced four days before he came into the hospital. Twenty ounces of blood were taken from the arm, and a draught of castor oil, oil of turpentine, and laudanum. 4th. Had a tolerably easy night. Was ordered mercurial ointment, to be rubbed on the thighs every four hours, and tartar emetic ointment to the spine every night— vapour bath—forty drops of laudanum every three hours. 6th. No alteration. 7th. Mouth sore—back covered with pustules—tetanic symptoms not increased. 9th. Severe pain at the pit of the stomach— castor oil draughts repeated, and operated. 10th. The spasms were frequent. He expressed a great desire for punch, of which he was allowed a moderate quantity. As the symptoms continued favourable, he was indulged with more of this beverage. 16th. All medicine was discontinued, and shortly afterwards he was discharged well.

It is evident that this was a mild case; but, whether the recovery can be attributed to medicine or nature is very problematical.

Of the five cases that died, only two were permitted to be opened.— In these, the spinal marrow and its sheath were examined, but no morbid appearances could be detected. In one of them, numerous contractions were observed in the ileum. After commenting on the bad success of the means hitherto employed in tetanus, Mr. Carmichael suggests (for he cannot rest on the basis of experience) the following:—

"From the general failure of the plans hitherto employed, I shall feel no hesitation (however hypothetical these views may appear to some) in giving a full trial in such cases of Tetanus as may next happen to come under my care, to alcohol in any of its various combinations— æther largely exhibited both in the form of draught and enema, and combined with opium—the tartar emetic ointment rubbed upon the abdomen, and at the same time promoting the action of the bowels by castor oil and turpentine." 292.

---

18. *Purpura Hæmorrhagica.** James Stodart, æt. 6, living in a confined part of the town, of a weak and strumous constitution, though lively, had been much confined to school, and had a swelling of the glands of the neck with inflamed eyes. On the 24th, and two following days of April, 1823, he appeared very dull, inclined to sit over the fire, with thirst and face flushed. On the 27th, spots, like flea-bites, some small and red, others larger and purple, appeared over a great part of his skin, and soon increased considerably. On the 28th, blood oozed from the mouth, with occasional bloody sputa. In the morning, the urine was red and turbid, and in the forenoon he walked a mile and a half, with his father, for medical aid. On the 1st of May, Dr. Gairdner

---

* Case of Purpura Hæmorrhagica. By Ebenezer Gairdner, M.D. Fellow of the Royal College of Physicians, Edinburgh; and one of the Physicians to the Royal Dispensary.—*Ed. Med.-Chir. Transactions, vol. i. p. 671, et seq.*

first saw the patient, and, from the symptoms, recognized immediately the purpura hæmorrhagica of Willan. The petechiæ, with vibices and ecchymoses, were numerous over the whole body, but crowded on the upper part of the back, breast, and anterior part of both thighs; some of the latter, about the size of a sixpence, of irregular shapes, were of a claret-colour; some felt rough, though not at all elevated. By careful examination through a powerful glass, the texture of the skin appeared quite entire. The conjunctiva of the right eye was ecchymosed, without œdema or lippitudo; there was neither diminution of sight, nor pain; tongue rather dry and strewed with several petechiæ; thirst; gums redder than usual, tender, and blood oozed from them; his breath fetid; hæmatemesis occasionally present; the Schneiderian membrane was streaked with blood, and at times it bled. Both hypochondria, particularly the left, were full and painful on pressure; abdomen rather tumid, with obscure pain; bowels costive; the urine, of a deep red, and turbid, was free, and often passed in sleep; skin nearly as usual; he seemed little oppressed, but attentive to questions.

A saline cathartic, and fifteen drops of the diluted sulphuric acid to be taken thrice a day: the warm bath, of about 80° Fahrenheit, evening and morning. *May 2nd.* Some sleep was obtained, though he had a bad night; breathing oppressed and quiet; hæmorrhagic symptoms increased; pulse 110 and wiry. He was bled, and when he had lost about ℥x. he vomited; no blood in the egesta, but he had spit clots in the night; pulse 124; skin hot; besides the bath and sulphuric acid, powders, containing calomel and jalap, three grains each, were ordered every three hours. On the 3d, the wound was not yet closed; blood had oozed from it since the operation; some vibices enlarged. All the symptoms were mitigated. On the 4th, the pulse 124; pain under the os frontis; ecchymosis of the eye greater; hypochondria more painful, with tension. Bleeding was again determined on, but, on removing the bandage, blood drained out, and the patient becoming faint with terror at the lancet, two or three ounces only were obtained. He sank into a sound slumber soon afterwards; pulse 124. *5th.* Urine pale and limpid; pulse 102; evacuations from the bowels black and offensive. The pain in the abdomen and hypochondria increased considerably, and castor oil was administered in small doses, with fomentations. Nature appearing inadequate to the removal of the disease, port wine and water, a pound of each, with an ounce of cinchona infused into it, was frequently given. *6th.* The pain in the bowels began to decrease, black fæces were still discharged, no oozing or spitting of blood, tongue natural, and the petechiæ appeared a little faded; pulse 98. He continued to improve daily till the 10th, when he was convalescent; the marks on the skin very pale, but the breath was fetid. On the 14th he was quite well.

The blood could not be analysed, as it was thrown away, but what was first drawn seemed, in four hours, to coagulate imperfectly into one mass. On the following day, it resembled a tremulous jelly, the top of a greenish-buff colour, interspersed with brownish spots. What was discharged afterwards was more like turbid lymph, or a fluid in which some reddish colouring matter was in suspension. The cloths were stained, as with dirty water, with spots of a reddish-brown hue. It is remarkable that the serum, by rest, undergoes a spontaneous, though slow coagulation.

"*Mr.* MURRAY'S *Analysis of the Urine.*

"No. I. voided on 2d May, at half-past 2 o'clock P. M. was of a brown colour, without smell. On standing, it deposited a precipitate of a darker colour, while the superior part of the fluid was of a dirty pale yellow colour, and turbid. When the urine was shaken, it resumed the original brown appearance; and a portion of it that was set aside, after eighteen hours standing, was still without smell.

"Exper. 1. Litmus paper was stained slightly red.—2. A dense white coagulum was produced by a heat of 180°.—3. Diluted nitric acid and alcohol produced a similar coagulum.—4. Corrosive muriate of mercury caused an abundant white precipitate.—5. Lime-water produced a very slight precipitate.—6. Potash did not cause any precipitate.—7. One fluid drachm contained $2\frac{1}{2}$ grains of solid matter; the urine contained $\frac{1}{24}$th part of solid contents.—8. A slight precipitate was produced in the clear liquid by the corrosive muriate and infusion of gall.—9. The clear liquid contained a considerable portion of a colouring matter, and but a small quantity of phosphoric salts, with no urea.

"Nos. II. and III. voided at 5 and half-past 5 o'clock P. M. of 2d May, differed principally from No. I. in containing more free acid and less albuminous matter, the urine containing only $\frac{1}{27}$th part of solid contents.

"In the 8th experiment, the precipitate was rather more copious, indicating the presence of gelatine and mucus.

"In the 9th experiment also, the appearances were rather more distinct.

"Dr. Combe has shewn, in his case of Purpura,* that there was an excess of albumen, with a deficiency of urea, similar somewhat, in this respect, to the present case." 680.

*Mr.* WOOD'S *Case.*

The patient, æt. 12, was scrofulous, having a chronic disease in her left wrist. June 21, a spot appeared on her under lip, like the mark from a pen, and the next morning, similar ones were seen studded over her legs and arms. She walked to Mr. Wood's house (about a mile's distance) with ease. Some opening medicine was ordered. Mr. W. found her next day sitting by the fire, her pulse good, and unconscious of ailment. Salts were given. At 10 at night, she had some milk and bread, and soon afterwards went to the water-closet, and again betwixt 3 and 4 A. M. when she was extremely faint and giddy. There

"* Edin. Med. Journ. No. 66."

was severe pain in the right temple, and distressing sickness supervened. What she vomited was tinged with blood, and her gums now bled readily. Most alarming languor and exhaustion succeeded; the tendency to vomit continued; the pulse was scarcely perceptible; symptoms of oppressed brain manifested themselves, and, at 3 P. M., she died comatose.

*Dissection.* Surface as before described. The pericranium and dura mater were covered with petechial spots. On removing the membrane, the effects of large effusion of blood were evident. In the right temporal region, a firm coagulum, floating in bloody serum, had forced its way through the broken-down brain into the ventricle. The serous membranes of the chest and abdomen were studded throughout with dark livid spots.

---

19. *Conversion of Disease.** The conversion of disease forms one of the most curious and perplexing problems in pathology. Dr. Crampton thinks that the fact of metastasis is now much doubted. It is so by a few young writers who can descant most learnedly, and pronounce dogmatically, on diseases and phenomena they never saw. But every man of even moderate experience and common attention, must be as well convinced of the occurrence of metastasis, conversion, transference, or whatever other name may be assigned to it, as of his own existence. That this curious process is an effort of nature or of the constitution, there can be little doubt. But it is not, therefore, a salutary process in all cases. Indeed the same remark may be applied to all Dame Nature's workings. They are often salutary—often fatal. There can be little doubt, however, that metastasis is a process which is, as it were, forced upon the constitution occasionally by the operation of rough or improper remedies. We agree, therefore, in the following sentiment of Dr. Crampton.

"Whatever difficulties may occur in attempting to explain these transitions, there is no doubt of the fact, that disease often becomes suspended in one part, and appears in another. Under judicious medical treatment, such occurrences are not very common; experienced physicians are prepared for them, and, in many instances, anticipate them; they are frequently averse to push medicines to their full extent of remedial power, least they may hazard such consequences; but where such conversions of disease are looked for, a cautious and more expectant treatment is oftener adopted." 70.

In the case about to be detailed, rheumatism was exchanged in succession for erysipelas, dysentery, peritoneal inflammation, and dropsy. In each successive change the preceding disease disappeared, and ultimately the patient was restored to health, after encountering considerable difficulties, as might be expected.

---

* Dr. John Crampton. Dublin Transactions, vol iv.

*Case.* M. R. aged 26, slender, and of fair complexion, after returning from harvest-work, in England, was admitted into Steevens' Hospital on the 2d October, 1820, complaining of pains in his wrists, elbows, shoulders, knees, and ancles. The knees were swelled and gave him most uneasiness. He had slight cough—pulse natural—no fever—skin dry. Attention was paid to the skin and digestive organs—and tepid baths were occasionally employed. Under this treatment the rheumatic affection subsided, excepting the swelling and pain in the knees. Leeches and fomentations were repeated three or four times, with an interval of a few days between each application. After three weeks residence in hospital, the tartar-emetic ointment was applied to the knees—but nothing appeared to make any impression on the local complaint. A blister was now applied to one of the knees. The pain deserted both knees. But presently the glands in the left axilla became enormously swelled and extremely painful—the surrounding cellular texture and skin inflamed; this appearance extending down the arm, the whole of which looked angry and erysipelatous, attended with symptomatic fever, delirium, rapid, small, and weak pulse—the powers of life appearing at a low ebb.

"Efforts were made to subdue the local inflammation by leeches successively applied, from the 3rd of December to the 10th, by cooling lotions and emollient poultices, by a strictly antiphlogistic regimen, with refrigerants, occasional opiates, and the necessary attention to the state of the bowels; but deep and extensive suppuration took place under the axilla, which exhausted the patient, and gave every reason to apprehend a hectic state. Wine, cordials, and a suitable diet, with appropriate surgical treatment, had, however, so far restored him, as to give reasonable hopes of recovery; but on the 17th of December disease again assailed him in a new and not less dangerous form." 73.

This form, or third attack, consisted in a painful state of the whole tract of intestines, attended with frequent liquid and bloody stools, indicating inflammation of the mucous membrane of the bowels.

Under existing circumstances, very active treatment could not be ventured on. Leeches to the abdomen and anus—castor oil—anodyne absorbent mixtures, were employed. The pain was mitigated, but the diarrhœa continued, notwithstanding a variety of remedies. At length it was determined to exhibit a grain of the superacetate of lead and half a grain of opium three times a day. The remedy was so far successful, that the disease soon assumed its fourth and last form.

"Ascites, with a painful and very distended abdomen was discernible, and little strength was now left upon which remedies of any considerable power could be brought to act. The peritoneum, at this period, was evidently inflamed, and effusion into its cavity was taking place as a consequence." 74.

Digitalis, blisters, leeches, and other appropriate means subdued at length the peritoneal inflammation and dropsical effusion.

"This case," says Dr. Crampton, "may be considered as an instance of that peculiar delicacy of constitution we often meet with in practice, where several organs are successively attacked with disease. Whether a scrofulous diathesis or other predisposition lays the foundation for such occurrences, it is not easy to say; but in the physician's intercourse with patients in the higher circles of society, often amongst females, when one disorder is nearly subdued, some new morbid train of symptoms arises. I recollect a case in an interesting female, where a smart pulmonic attack first claimed attention; on the subsidence of this, an hepatic inflammatory disease ensued; in succession, a nephritic one. When relieved in one quarter, the same morbid circle of disease was renewed, so that it was nearly impossible to restore her to health. With such a description of patient, moral causes have also their influence, and must be taken into account by the physician." 76.

Was the first metastasis, or transference of inflammation from the knees to the axillary glands and cellular membrane, occasioned or *accelerated* by the blister? We think the *latter* very probable, and so does Dr. Crampton. In the following passage, we almost entirely agree with our ingenious author, and we recommend the passage to the attention of the reader.

"The last change, as to the parts affected by disease, is more readily understood. The transition was merely from the mucous to the serous coats of the intestines and the peritoneum. That the superacetate of lead was in some degree instrumental in effecting this conversion, there is, I believe, little doubt; but it was a change on the whole which improved the patient's chance of recovery. A remedy possessing so high a degree of astringency soon arrested the profuse discharges from the inner coats; the outer, or serous coats, then became the seat of irritation. The peritoneal inflammation, although occurring in a debilitated subject, no time being lost, gave way, partly to the treatment adopted, and partly to its natural cure by effusion of serum into the abdominal cavity or dropsy. Finally, the dropsical effusion was disposed of, by using remedies to subdue inflammation, to promote absorption, and to diminish exhalation." 78.

We said we *almost* entirely agreed with our author in the above passage. We cannot quite coincide in his idea that *peritoneal* inflammation and effusion into the abdominal cavity was a condition preferable to inflammatory affection of the *mucous membrane* of the bowels and consequent diarrhœa. We certainly should prefer the latter condition, were it in our choice, whether to treat it in others, or suffer it ourselves.

---

20. *Effects of Lightning.** On the 16th of April, 1812, the Coldstream East-Indiaman, being near the line, was struck by lightning twice, at an interval of about 15 seconds. The effect upon the vessel

* Cases of Persons struck by Lightning. By Alex. Macaulay, M.D.—Ed. Med.-Chir. *Transactions, vol.* i. *p.* 360, *et seq.*

was dreadful. Marks of fusion appeared in a brass pulley at the top of the main-mast, which was struck spirally and greatly shattered;—a lascar, who was standing at the foot of it, was killed upon the spot. Another lascar was rendered insensible, but recovered in a quarter of an hour. Three cases of severe injury occurred.

*Case* 1. George Brown, gunner's mate, was picked up in a state of complete apoplexy: his countenance livid and bloated, his body covered with a cold, clammy sweat: breathing anxious and stertorous; pulse slow, strong, and full; and sense and volition suspended. Fifty ounces of blood were taken in full stream, which removed the stupor and other alarming symptoms. This was followed by purging and antiphlogistic regimen. In about three days, there was much pain in the region of the liver, which was dissipated by the application of a blister. He gradually recovered.

*Case* 2. Samuel Cramp, seaman, in the main-top when the ship was struck, was able to come down to his berth, but was soon attacked with very violent epileptic fits. These were always preceded by his imagining that he saw coming towards him a spectre-sailor, (respecting whom a story had got about.) Bleeding was largely employed. The febrile symptoms continued for several days, and, on the fourth, he appeared suddenly to have lost his voice, which, however, he recovered in two days. He said that speaking gave him acute pain in the right side. He recovered, but, when intoxicated, he was ever after louder and more turbulent than before the accident.

*Case* 3. A soldier, in the St. Helena regiment, was struck. He too had epilepsy, which was stopped by depletion; the fits continued to recur for several weeks afterwards. He was subject to epilepsy, but had not been attacked during the voyage, previous to the thunder-storm.

All the cases, the author remarks, shewed evident marks of determination to the head. It is extremely probable that the two last patients were influenced more by the passion of fear, than by the lightning. Dr. Gregory used to give as an instance of what Cullen calls apoplexia mentalis, the case of an elderly lady, who was much terrified by very vivid lightning, complained of head-ache, which went on to apoplexy, and proved fatal in thirty-six hours. The pain in the region of the liver, noticed in two cases, is remarkable.

---

21. *Inflammation of the Appendix Cœci.** There are some cases of this kind on record before M. Vilermay's. This gentleman has recently published two instances.

The *first* was a man of sober habits and good general health, who was suddenly attacked, on the 18th November, 1823, with acute pain

* M. Vilermay. Archives Generales.

right side of the abdomen, followed by vomiting. The testicle side was retracted—pulse little altered. Emollients and 20 leeches to the anus. Better in the evening; but the night was restless, the pulse was quickened. *Second day*, 30 leeches to the right iliac region, the seat of pain. Sixteen ounces of blood were also taken from the arm. *3d day*, 20 leeches to the seat of pain—warm bath every six hours—vomiting in the night of dark green and blackish matters. *4th day*, pulse gone, extremities cold. Expired at mid-day.

*Dissection.* In the right iliac fossa there were five ounces of black serum effused, emitting a gangrenous smell. In the midst of this floated the appendix vermiformis gangrened. The gangrene did not extend to the neighbouring parts. The cellular tissue surrounding the right kidney was infiltrated with a sero-purulent liquid, the gland itself being free from disease. The mucous membrane of the stomach was red and thickened.

*Case 2d.* A man had an inguinal hernia of the right side, which he kept accurately reduced, by means of a truss. After supper (21st July) he was seized with desire to vomit, and a violent pain near the umbilicus, with a sensation of a bar crossing him there. He brought up some green viscid bile—the pain and tension increased. 22d. At six this morning there was a calm, but attended with extreme debility, pallor of the countenance, thirst, small irregular pulse, cramps in the calves of the legs. Venesection—leeches—fomentations ordered, but not complied with. At 2 P. M. green vomiting, dysuria, great tumefaction of the abdomen, rigors. Subdelirium in the night. 23d. Died at 10 o'clock this morning.

*Dissection.* The appendix vermiformis was observed to be three inches long, and an inch in diameter at its base, of a bluish-black colour. When opened from its external side, the cæcum was found to be healthy even as far as the mouth of the appendix. The intestines were healthy, but the mucous membrane of the stomach was inflamed.

Inflammation of the caput coli itself is by no means an uncommon disease, and is almost invariably occasioned by hardened fœcal matters or indigestible substances lodged there. We have met with many cases of this kind, that caused great constitutional disturbance and some danger of life. After local, and if necessary, general bleeding, we have found a considerable dose of opium combined with a cathartic, and, in a few hours, followed by repeated purgatives, the best means of dislodging the offending matters.

---

22. *Partial Intermittent Fever.** Intermittent diseases present the most inexplicable phenomena of any class of human ailments. They are the rock on which *systems* split—while they are the glory of *practical medicine*. *Les fièvres intermittentes sont devenues l'écueil des sys-*

* Fh. Des-alleurs. Revue Med. Mai, 1824.

*tèmes—elles sont la gloire de la médecine d'observation.* Observation would seem to prove that, whereas a regular intermittent affects, during its paroxysms, the whole system, there are numerous other forms of the same disease which fix their seat in a particular part, or point, as it were, of the body, leaving all other organs and parts free. The following is a curious example.

A woman, aged 45 years, who officiated as a nurse, and was of a nervous temperament, complained to our author of an affection of the right arm, to which she had been subject for more than a year. It began with a sense of numbness and tremor of the extremity, which sometimes lasted two hours or more, then a sense of heat occurred—and finally a perspiration broke out over the arm, the whole paroxysm lasting some hours, and leaving the patient in a languid condition for a few hours more, after which she felt quite well, till another paroxysm took place. The intervals, at first, were three or four days, but latterly they have returned at shorter periods. She had tried a multitude of remedies, without receiving the least benefit. Our author first gave emetics, followed by bitter and antispasmodic drugs, but to no purpose. The appetite was somewhat improved, but the local affection remained. He now requested to be present during one of the attacks, and found as follows: the patient was sitting, her countenance somewhat depressed, but, otherwise, apparently in her ordinary condition. Her right arm, though well covered and near the fire, felt to herself as cold as ice—Its temperature was not much decreased to the tact of others, except the hand. A tremor of the limb now came on, and was pretty violent. The pulse was nearly natural. In half an hour the arm became red, and the sensation of cold went off, changing to that of intense heat, though still but little altered in real temperature, till a quarter of an hour had elapsed, when the heat was very sensible to the touch of the bystander. In half an hour more the whole extremity was covered with perspiration—the patient's spirits rose, and in the course of another hour the paroxysm was over. At the close of the attack the patient passed a pint of high-coloured urine, cloudy, but not sedimentous.

Our author now became convinced that the disease was a local or partial intermittent, and determined to treat it accordingly. The bark was therefore administered. It first had the effect of mitigating, and ultimately, of entirely stopping the paroxysms. The patient has since continued in good health.

---

23. *Animal Putrefaction capable of producing Fever resembling Plague.** We had lately an opportunity of shewing how erroneous was the dogma of Dr. Bancroft and his partisans that the fevers of hot climates (yellow fever so denominated) never became contagious in their progress. Another dogma which he has maintained with more forensic argument than matter-of-fact evidence, is, that *animal matters*, in a state

* Private but Authentic Information.—*Editor.*

of putrefaction, never produced fever. The following authentic fact will not tend much to the stability of the learned Doctor's theory.

An American merchant ship was lying at anchor in Wampoa Roads, 16 miles from Canton. One of her crew died of dysentery. He was taken on shore to be buried. No disease of any kind had occurred in the ship from her departure from America, till her arrival in the River Tigris. Four men accompanied the corpse, and two of them began to dig a grave. Unfortunately they lit upon a spot where a human body had been buried about two or three months previously (as was afterwards ascertained.) The instant the spade went through the lid of the coffin, a most dreadful effluvium issued forth, and the two men fell down nearly lifeless. It was with the greatest difficulty their companions could approach near enough to drag them from the spot, and fill up the place with earth. The two men now recovered a little, and with assistance reached the boat and returned on board. On the succeeding morning they were visited by an assistant-surgeon from an English Indiaman in the roads, who reported the following symptoms: viz. very acute head-ache, with a sense of giddiness, and dimness of sight (which had existed more or less from the moment of opening the grave) eyes of a peculiar muddy appearance, "resembling that generally observed in cases of Indian cholera"—oppression about the præcordia—dull heavy pain in the regions of the heart and liver, with slight palpitation at times, and fluttering pulse—sense of extreme debility, with occasional convulsive or spasmodic twitchings of the muscles of the lower extremities—nausea—slight diarrhœa—rigors succeeded by flushings of the face, neck, breast, and upper extremities—tongue white and much loaded—pulse from 110 to 120, weak and irregular—urine scanty and high-coloured—skin sometimes dry, sometimes covered with a clammy sweat. On the fourth day from the commencement of the attack, numerous petechiæ appeared over the breast and arms—and in one of the patients, a large bubo formed in the right groin and another in the axilla of the same side, which speedily ran on to suppuration. To one, the disease proved fatal on the evening of the fourth day—to the other, on the morning of the fifth. For two days previously to death, the gums bled freely.—The symptoms were so completely similar in both the cases, that it is needless to repeat them here. Purgatives, venesection to fifteen ounces, blisters—and ultimately stimulants, were the means ineffectually had recourse to in these two cases.

*Dissection.* Mr. Hamilton, surgeon of his Britannic Majesty's ship Britomart, was present at the post mortem examination. There are few medical officers in the army or navy better qualified to appreciate the appearances.

In both cases, the vessels of the brain were loaded, and an effusion of lymph existed between the tunica arachnoidea and pia mater—a more than usual quantity of fluid in the ventricles—upwards of three ounces of a dark-coloured liquid at the base of the brain. The optic nerve, on the right side, of one of the patients, was surrounded by a portion of gellatinous matter, where it emerges from the thalamus, and appeared thickened and discoloured—surface of the brain very vascular. The heart, in both cases, was much enlarged, and distended with blood. In one case, five ounces of a dark-coloured fluid flowed from the pericardium when slit open, and the vessels on its internal surface were gorged with blood. The liver was enlarged in both instances, and its vessels completely gorged with blood, and this was indeed the case throughout the whole portal circle. The stomach, near its pyloric orifice, was thickly beset, in one case, with small purple-coloured spots. The intestines in several places assumed a brownish appearance, as likewise the omentum. There were numerous petechiæ on the surfaces of both bodies. In one of the cases, the medulla spinalis, in the dorsal region, to the extent of three or four inches, was of a light brown colour; and in the other case, it exhibited evident marks of congestion throughout. Most of the inguinal and axillary glands were enlarged and hardened, and several of them, when cut into, contained a light straw-coloured matter. No other morbid alteration of structure existed.

One of the two, not immediately engaged in digging the grave, was attacked on the eighth day from his being on shore, and Mr. Hamilton was requested to attend with the other medical gentlemen. They found the patient retching violently, and labouring under all the symptoms of the former patients, in an aggravated degree. On examination, it was found that there had existed, for three days previously, pain and enlargement of one of the inguinal glands, which had now acquired the size of a hen's egg. For that space of time he had also felt indisposed, but did not complain or abstain from duty. Notwithstanding the state of the pulse, which was very weak and irregular, Mr. Hamilton immediately bled the patient to the extent of twenty-five ounces, previously placing him in a horizontal posture, with the view of preventing too early syncope. Twenty grains of calomel and one fluid drachm of tincture of opium were administered instanter. This, with some other minor means, had the effect of allaying the gastric irritability. The pulse rather increased in strength during the bleeding;—but soon afterwards became almost imperceptible at the wrists. Stimulants of ether, brandy, &c. were now exhibited—the patient was stripped of his clothes, and a flannel gown, previously moistened on its inner surface with a mixture of brandy and nitric acid, was put on. By means of a portable vapour bath, steam was now applied to the body and continued for half an hour, when so considerable a reaction came on, that Mr. H. thought proper to restrain it by means of another bleeding to the amount of twenty-five ounces. This had the desired effect of relieving a very acute head-ache, and severe giddiness, as well as a sense of fulness and dull heavy pain in the regions of the heart and liver. Upon removing the flannel gown, the whole surface of the body appeared highly inflamed and tender to the touch, and the petechiæ which had previously been spread over the breast and arms, had now almost wholly disappeared. Being enveloped in a blanket, he was now removed to bed. The liq. ammon. acet. and camphor were given as sudorifics, and calomel and jalap to open the bowels. M. H. visited him in six hours after he was put to bed—and had the mortification to find him in as bad a state as when first seen.—The pulse was very feeble—surface covered with clammy sweat—lower extremities cold. The bath and other means before adopted (with the exception of venesection) were again employed, and reaction was once more excited, together with free perspiration. An alleviation of the symptoms was the consequence. Friction with mercurial ointment and liquor ammoniæ was directed over the abdomen and thighs, while calomel was freely given until the system came under the influence of mercury, gentle purgatives being administered the while. Venesection was a third time necessary to moderate excitement and promote the action of the mercurials. The enlarged gland in the groin did not run on to suppuration, but continued hard and the same size as at first.—On the fifth day, this patient was out of danger, being in a state of ptyalism. It was about three weeks before he was able to resume his duty. The fourth man had a slight indisposition; but not of any consequence or decided character. Every precaution was taken to prevent the diffusion of the disease on board the vessel, by cleanliness, ventilation, fumigation, white washing, &c. and no farther sickness occurred.

We think the foregoing document (from official records) will satisfy the most sceptical that putrid *animal* matters, may, under certain circumstances, produce fever, and that of so malignant and fatal a character as to closely resemble plague itself. No little credit is due to Mr. Hamilton and the other medical officers, for the zeal and ability manifested in the dissection of the fatal cases, and treatment of the man who survived.

---

24. *Pathology of Epilepsy.** About eight years ago, Dr. Reid's attention was drawn to the subject of epilepsy, when attending the hospitals with the late Dr. E. Percival. Spirit of turpentine was then strongly recommended in epileptic diseases. While studying the phenomena of this formidable disorder, our author felt convinced, that an extensive field for the investigation of its pathology was still open. In epilepsy, it is evident, that a derangement of the moving powers forms its most prominent characteristic feature—and as these moving powers are under the influence of the nervous mass situated in the spinal column, it ought to be ranked, our author conceives, among those diseases to which the spinal system is liable.

Pure epilepsy, Dr. Reid observes, is seldom fatal. In 34 cases which came under his notice, two only died of the disease. On examination of these, *post mortem*, no morbid appearance, sufficient to account for death, could be found till the spinal column was opened. Within the cervical vertebræ the membranes enveloping the medullary mass appeared covered with a "minutely injected vascular tissue."

"The morbid states of other parts, particularly in the head, which have been the immediate cause of death in cases previously subject to epilepsy, can only be considered the consequences, for similar states

* Dr. Robert Reid. Dublin Transactions, vol. iv. p. 354, *et seq.*

have been met where no such disease attended. It is important to be aware, when investigating the diseases incidental to the spinal system, that they frequently run into each other. Thus a person while under treatment for Epilepsy may suffer tetanic spasm, then chorea, then catalepsy, &c.: a case of this kind occurred to me not long since, of which the following is an abstract." 356.

This case we shall considerably abridge.

A young gentleman, 18 years of age, was subject for several years to epilepsy; but with no malformation of the head. The fits vary in frequency from thrice a day, to twice a week. Has no aura epileptica—pulse contracted, quick, and irritable. Our author attended him at the expected hour of an attack, and found that the pulse ceased at the wrists for some minutes before the paroxysm. This he found to be the prelude of a fit in other cases. A blister kept open on the spine for a short time seemed to diminish a little the frequency of the seizures. Repeated doses of oil of turpentine, however, had much more effect in reducing the returns of epilepsy.* The medicine produced copious evacuations from the bowels, and considerable irritation in the urethra, till even some drops of blood were passed after making water. The quantity was then reduced to five drops every morning. But he could not be induced to continue the medicine, and the fits returned with their usual frequency, and his mental powers became rapidly impaired. *August* 1. Is delirious at intervals this evening. Complains of pain in the course of the nerves of the lower extremities, with frequent sensations of cold along the spine, sometimes ascending to the head. *August* 31. Has frequent tetanic spasms. The cerebral symptoms have been relieved by leeches and cold affusion to the head. Perspires copiously—pulse quick but soft—tongue white in the centre. *Sept.* 1. Has had slight cramps in the lower extremities, and is often affected with convulsive laugh. Determination to the head—leeches, cold affusion, &c. *Sept. 3d.* Skin soft and moist—hearing morbidly acute—sight improved—memory and other intellectual powers natural. *Sept. 4th.* Feels well. He continued convalescent till the middle of September, when he went to the country for change of air.

Dr. Reid has selected the foregoing case as remarkable for the number and variety of the changes from one spinal disease to another. In all the cases our author has had an opportunity of observing, "the rapidity in the changes of the morbid actions, during the paroxysm, often differed very much; *but the regular succession in which they followed each other was invariable.*" It appears to Dr. Reid, that each succeeding occurrence, is the natural consequence of that immediately preceding. The first symptom of attack, he avers, is the suspension of the pulse, varying in duration from a few seconds to about three minutes—the

* It is unpleasant to see contradictions in a plain statement of facts.—Dr. Reid avers, that the patient had no fit between the 26th June and 18th July, after stating in the very paragraph preceding this, that he had a fit on the 1st July.—*Rev.*

longest period he has yet known. The *aura epileptica* is uncertain, for in many cases it does not occur at all.

"If all the other powers which contribute to circulate the fluids in the animal frame became quiescent at the time the heart ceased its activity, the well-known phenomena of fainting would be the consequence. But, during the epileptic quiescence of the heart, it appears that an accumulation of blood is taking place in another direction; for immediately, often instantaneously, a tetanic rigidity of the entire frame succeeds, during which the air is sometimes so suddenly expelled from the lungs, that the patient utters a piercing shriek. I have seen patients hop five or six times on both feet, with their bodies perfectly rigid, before they fell. It has been attempted to prove in the Treatise before referred to, that the tetanic rigidity of all the muscles of the frame was consequent upon accumulation of blood, or other irritation in the spinal nervous mass.—That this is the case in the present disease is confirmed by the examination of those cases which have proved fatal after only a few paroxysms, and before the injurious effects of the disease were conspicuous in other parts. When this accumulation amounts to compression of the nervous structure, the patient falls, and all the parts are for a moment relaxed.—The animal economy now imperiously requires the function of respiration, which had been suspended during the phenomena before-mentioned; but this suspension of respiration was gradually causing an accumulation of blood in the head, so as to bring the brain into an apoplectic state, by which means the control of the brain over muscular action is interrupted. Hence arise the irregular actions of the complicated organs of respiration, forming those convulsive struggles which are so peculiarly characteristic of this disease." 362.

We confess that to us there appears much fanciful supposition in the foregoing theory. We see no proof whatever, that, upon the suspension of the heart's action, there should be an accumulation of blood in the spinal nervous mass, more than in other parts. Why should not syncope, in other cases, produce the phenomena of epilepsy? What right have we to suppose that the *other moving powers* of the circulation go on during the *epileptic* quiescence of the heart, but cease in common fainting? We do not believe that the arteries cease to contract and go on propelling the blood in syncope—and for this reason, that we know they do propel it into the veins after death itself, which must surely be the greatest degree of syncope.

We now proceed to state a fact—at least, Dr. Reid asserts it to be a fact—although we are very far from agreeing with our author in his mode of accounting for the said fact. This is, the immediate termination of the paroxysm by mechanical means. These means are two-fold. One method consists in forcibly extending the hands and opening the fingers of the patient;—the consequence of which is, that—"so much exertion is involuntarily made by the patient to oppose this, that the violent operation of the respiratory muscles subsides, the organs fall into their natural train of action, the patient draws a heavy sigh, and the paroxysm is at an end." The other mode of stopping the paroxysm is still more powerful, in our author's experience. It is to be accomplished —" by pressing the closed hand of an assistant forcibly on the soft part of the abdomen, towards the spine, while the patient is firmly supported on the back, with the head and shoulders raised."

We have said, that in admitting the facts, on the testimony of Dr. Reid, we were not inclined to subscribe to the doctor's explanation of them. It is but fair, however, to let him explain himself.

"When making experiments some time ago, for the purpose of ascertaining what part of the animal frame was particularly acted on by nux vomica, when taken in excess, I found that the animals (rabbits and dogs) in a short time after receiving the poison into their stomachs became tetanic. During the spasm I observed that the peritoneum seemed closely to invest and compress the contents of the abdomen. Upon pressing forcibly a part of this membrane between my fingers, for the purpose of detaching one portion of it, so as to relieve the supposed compression of the bowels, I was rather surprised to find the spasms totally relax, and the animal begin to breathe, as if recovering from much fatigue. The moment the peritoneum was let loose, the spasms returned with violence; and this could be repeated at pleasure."* 364.

Reflection on the foregoing phenomena led our author to the two modes of arresting the epileptic orgasm, which we have just detailed. It is certainly of comparatively little consequence how the facts are accounted for—provided they *are facts*—and we hope they will be proved such by the experience of others. Considering how much mischief is done by protracted epileptic paroxysms, we think, Dr. Reid will be entitled to the thanks of the profession if his modes of shortening these convulsions of nature be verified by general experience.

---

25. *Liver Cough.*† In the number for December, 1821, of this Journal, we stated the particulars of an interesting case of sympathetic (or as Dr. Brooke insisted of *liver*) cough, on which we took the liberty of giving our opinion that the evidence of enlargement of the liver was defective, while the existence of a collection of fæces in the colon was indisputable. Dr. Brooke maintains his opinion still that the disease was enlargement of the liver—and the best answer we can make him is to refer him to Mr. Callow's case of supposed enlargement of the liver, reviewed in Number 1 (New Series) of this Journal, for June last. In the relapse of the young gentleman, detailed in the recent volume of the Dublin Transactions, we see as little proof as before of hepatic dis-

---

* "It may be worthy of observation, that after repeating the above experiment of alternately compressing and letting go the peritoneum several times, the animal at length ceased to be affected by spasm, although upon killing it the poison was found still remaining in the stomach and small intestines." 364.

† Dr. Brooke. Dublin Transactions, Vol. iv.

It is true mercury cured the cough—but surely Dr. B. will not insist upon this circumstance as proof of diseased liver. It appears that the young gentleman had been living imprudently in respect to food, and had got cold in travelling up from the country.

"On the first of February he awoke, and found with dismay that the well known *bark* had returned precisely as before. I saw him in the course of the day, and soon recognized the harsh, loud, and monotonous cough. It was not perhaps so severe or frequent as last year, but was, indeed, very distressing; pulse 70, and rather weak; breathing perfectly regular; skin cold; tongue white, but quite free from fur; belly rather drawn in than tumid, but soft, and without any pain on pressure. He reported his bowels as regular, and his urine as being in small quantity, and high coloured.

"I could not discover any enlargement of the liver, or tenderness in the side. Conceiving, however, that the cough might arise from irritating fæces somewhere pent up in the intestinal canal, I ordered an active saline purgative. Nothing extraordinary appeared in the evacuations, and the patient only felt weakened by its effects, and expressed his belief, founded on former experience, that mercury alone could afford him relief. My conviction being quite in unison with his feelings, I prescribed on the following day a drachm of blue pill, with half that quantity of gum ammoniac, to be formed into twenty-four pills, two each night, and one each morning. His appetite was indifferent, and, with the exception of solid animal food and wine, he was allowed to live as he wished. He continued on this plan for a fortnight, without any apparent change. As before, he coughed the whole day, and passed the night in tranquil sleep; bowels daily regular, but the evacuations unhealthy in both appearance and smell; the urine continued crude and scanty." 146.

After some medicines had been tried without success, mercury was pushed till the mouth became affected, and the cough disappeared.

We have no doubt but the cough, in this instance, as also in the preceding year, was symptomatic of some abdominal irritation; but still we adhere to our former opinion that there is no proof of actual disease of the liver. That its function might be disordered, as well as the functions of the stomach and bowels, we grant. But this is a very different thing from what is meant by enlargement or organic disease of the liver. We could offer some strictures on Dr. Brooke's pathological observations at the end of the case; but we leave them to the consideration of the reader.

